# Endocrine-disrupting chemicals and the risk of gestational diabetes mellitus: a systematic review and meta-analysis

**DOI:** 10.1186/s12940-022-00858-8

**Published:** 2022-05-16

**Authors:** Dandan Yan, Yang Jiao, Honglin Yan, Tian Liu, Hong Yan, Jingping Yuan

**Affiliations:** 1 Department of Pathology, Renmin Hospital of Wuhan University, 238 Jiefang-Road, Wuchang District, Wuhan, 430060 People’s Republic of China; 2 Department of Health Toxicology, MOE Key Lab of Environment and Health, School of Public Health, Tongji Medical College, Huazhong University of Science and Technology, 13 Hangkong-Road, Wuhan, 430030 People’s Republic of China

**Keywords:** Endocrine-disrupting chemical, Gestational diabetes mellitus, Meta-analysis, Systematic review, Risk factor

## Abstract

**Objective:**

To conduct a comprehensive systematic review and meta-analysis to estimate the relationship between endocrine-disrupting chemicals (EDCs), including polychlorinated biphenyls (PCBs), poly-brominated diphenyl ethers (PBDEs), phthalates (PAEs), and per- and polyfluoroalkyl substances (PFAS) exposure and risk of gestational diabetes mellitus (GDM).

**Methods:**

Relevant studies from their inception to November 2021 were identified by searching EMBASE, PubMed, and Web of Science. The cohort and case–control studies that reported effect size with 95% confidence intervals (CIs) of EDC exposure and GDM were selected. The heterogeneity among the included studies was quantified by *I*^*2*^ statistic. Publication bias was evaluated through the Begg and Egger tests.

**Results:**

Twenty-five articles with a total of 23,796 participants were found. Results indicated that exposure to PCBs has a significant influence on the incidence of GDM (OR = 1.14; 95% CI = 1.00-–1.31; *n* = 8). The risk of GDM was found to be associated with PBDE exposure (OR = 1.32; 95% CI = 1.15–1.53; *n* = 4). PAEs and PFASs exposure were also positively associated with the risk of GDM, with summary ORs of 1.10 (95% CI = 1.03–1.16; *n* = 7 for PAEs) and 1.09 (95% CI = 1.02–1.16; *n* = 11 for PFASs), respectively. When only cohort studies were considered, the summary OR between PCBs exposure and the risk of GDM was 0.99 (95% CI = 0.91–1.09; *n* = 5). Meanwhile, the summary ORs from cohort studies for PBDEs, PAEs, and PFASs exposure were 1.12 (95% CI = 1.00–1.26; *n* = 2), 1.08 (95% CI = 1.02–1.15; *n* = 5), and 1.06 (95% CI = 1.00–1.12; *n* = 8), respectively. The Beggs and Egger tests did not show publication bias, and the sensitivity analyses did not change the results in this meta-analysis.

**Conclusion:**

These results support that exposure to certain EDCs, including PCBs, PBDEs, PAEs, and PFAS, increase the risk of GDM. Further large-sample epidemiologic researches and mechanistic studies are needed to verify the potential relationship and biological mechanisms. These results are of public health significance because the daily EDC exposure is expected to increase the risk of GDM development.

**Graphical Abstract:**

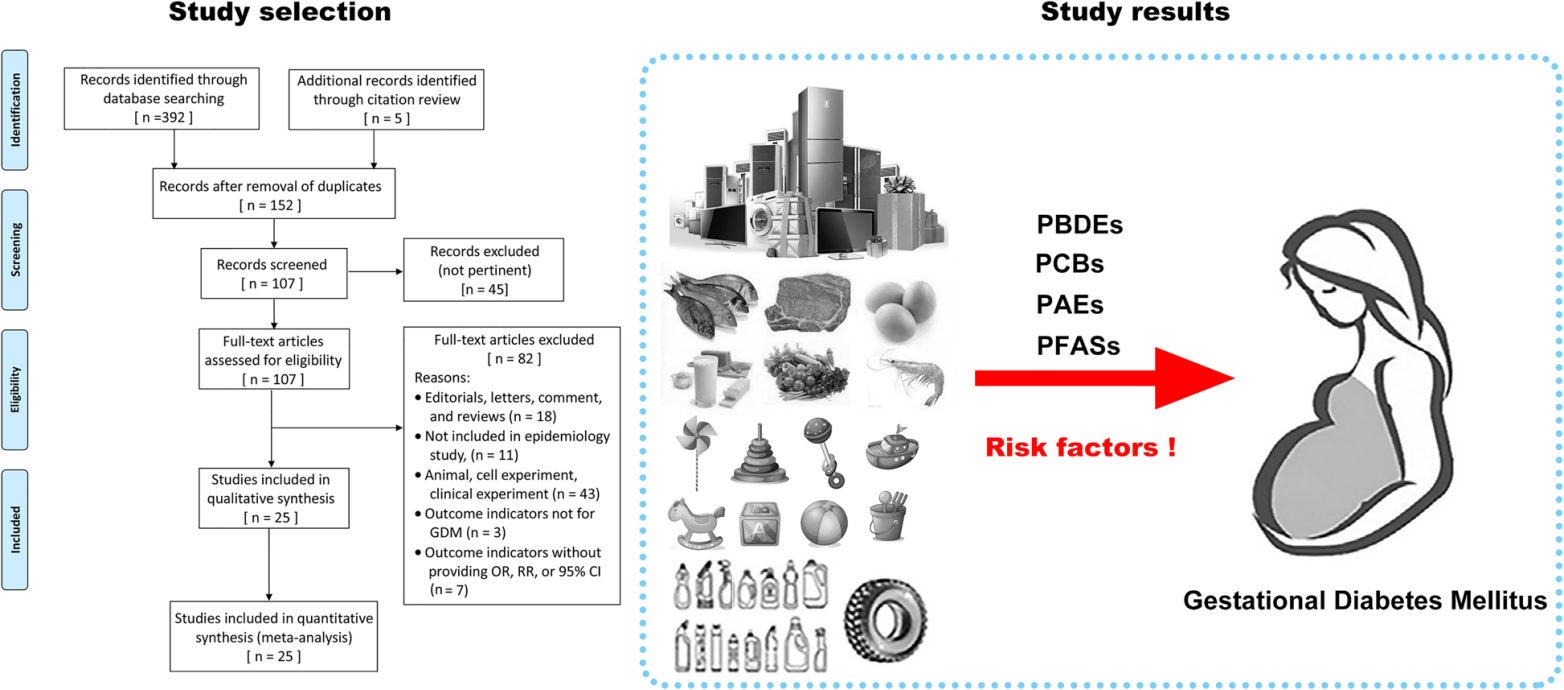

**Supplementary Information:**

The online version contains supplementary material available at 10.1186/s12940-022-00858-8.

## Introduction

Gestational diabetes mellitus (GDM) is one of the most common pregnancy complications [1], and it is diagnosed in the second or third trimesters of pregnancy when there was no overt diabetes prior to gestation. The incidence of GDM widely varies depending on the diagnostic criteria used and population characteristics [2, 3]. The prevalence of GDM is increasing, which is linked to an increase in maternal obesity in recent decades, and it affects 6% – 25% of pregnant women (depending on the diagnostic criteria) [3]. According to the 9^th^ edition of the International Diabetes Federation Diabetes Atlas 2019, 20.4 million women worldwide suffered from hyperglycemia during pregnancy, with 83.6% of them were diagnosed with GDM [4]. Evidences indicated that GDM is associated with dramatic adverse health effects for the mother and their offspring. Fetuses born to mothers with GDM are at an increased risk of multiple complications, including macrosomia, birth injury, altered metabolic status, neonatal hypoglycemia, respiratory distress, and type 2 diabetes mellitus (T2DM), later in life [5–10]. Meanwhile women with GDM are more likely to develop gestational hypertension, preeclampsia, caesarean section, and shoulder dystocia, among other serious complications [5, 6, 9, 10]. Maternal characteristics, such as advanced age, ethnicity, high-carbohydrate diets, pre-pregnancy obesity, and family history of T2DM, have been proven to be associated with an increased risk of GDM [9, 11]. However, the exact reasons behind the GDM are still unknown because over 50% of GDM patients do not have these classic determinants, suggesting the potential role of environmental factors [12].

Endocrine-disrupting chemicals (EDCs) are a special type of exogenous chemicals that can interfere with considerable normal endocrine signals [13]. These checmicals are structurally similar to some endogenous hormones that can disturb the synthesis, secretion, or elimination of natural hormones, which may result in hormonal disruption, including obesity, diabetes, and developmental dysfunctions [13]. Moreover, EDCs are widely existed in food packaging, medical equipment, personal care products, fabrics and upholstery, water, ambient air, detergents, and many industrial products [13]. The use of EDCs has significantly increased in the last few decades. Humans are frequently exposed to various EDCs from diet, household or agricultural pesticides, and cleaning products in everyday life. The pervasive use of EDCs and their association with chronic diseases have raised potential human health concerns and considerable public health problems [14].

Several EDCs, including polychlorinated biphenyls (PCBs), poly-brominated diphenyl ethers (PBDEs), per- and polyfluoroalkyl substances (PFAS), and phthalates (PAEs), have been reported to be associated with impaired glucose metabolism and T2DM [15, 16]. Growing evidence suggests that human exposure to EDCs is particularly concerning during pregnancy, as the effects of which on the developing fetus may result in long-term postnatal pathologies, such as intrauterine growth restriction and preeclampsia [17]. Previous studies have indicated that exposure to EDCs, including phthalates, bisphenol-A, and metals, may be closely associated with the occurrence and development of GDM [18, 19]. For instance, some studies indicated that exposure to PAEs may be correlated with the occurrence of gestational impaired glucose tolerance [20] and GDM [21]. However, epidemiological evidence for the relationship between EDCs and GDM is inconsistent due to the varying adjustment model variables, such as population selection, measurement approaches, and definition of outcome events.

Humans are widely exposed to various EDCs, which may influence the regulation of glucose homeostasis. However, the current studies linking EDCs exposure to glucose homeostasis during pregnancy were inconsistent and have not been well systematically reviewed. This study aims aimed to conduct a comprehensive systematic review and meta-analysis to evaluate the relationship between EDCs exposure and risk of GDM. The objectives of this study are as follows: (i) to confirm the association between EDCs and the risk of GDM and provide up-to-date epidemiological clues on this association, and (ii) to determine the pooled data on different types of EDCs (including PCBs, PBDEs, PAEs, and PFAS) and their influence on the risk of GDM.

## Methods

This meta-analysis was performed according to the guidelines of the Preferred Reporting Items for Systematic Reviews and Meta-Analyses (PRISMA) [22] and the Meta-analysis of Observational Studies in Epidemiology (MOOSE) [23]. The PRISMA checklist for this study can be seen in Additional file 1. This study is registered with the PROSPERO registration number CRD42021226856.

### Data sources

A comprehensive electronic search was carried out in the EMBASE, PubMed, and Web of Science databases for relevant studies from their inception to November 2021. The search terms included exposure (endocrine disruptor OR endocrine disrupting chemicals OR environmental pollutants OR persistent toxic substance OR polychlorinated biphenyls OR phthalates) and outcomes (gestational diabetes mellitus). The detailed search strategy is presented in the Additional file 2. We also checked the references of relevant articles to search for additional studies.

### Study selection

The titles and abstracts of the search results were independently screened by two investigators (DDY and YJ) according to the following inclusion criteria: (1) observational epidemiological studies (i.e., cohort, cross-sectional, or case–control studies) on the relationship between EDC exposure and risk of GDM; (2) the level of EDC exposure in humans is determined in biological samples (plasma, serum, or urine); (3) the outcome data reported the effect size with 95% confidence interval (CI) or sufficient data to calculate the effect size and 95% CI; and (4) provided data on sub-group EDC exposure, which have been studied in at least three studies; thus, the extracted data can be integrated. Studies that detected the level of EDCs through questionnaires or environmental measurements were excluded. Reviews, editorials, letters, and nonhuman studies were excluded because they could not provide the effect size and 95% CI on the correlations between EDCs and risk of GDM. The studies whose results could not be extracted or results could not be translated into odds ratio (OR) or 95% CI were also excluded.

### Data extraction and quality assessment

Data extraction was independently performed by two researchers (DDY and YJ) using the standardized data extraction sheet. The detailed data extraction sheet included the following items: first author, year of publication, location, study time period, design, sample size, covariate adjustment, diagnostic criteria for GDM, effect sizes, and 95% CIs. The comparison value between the highest versus the lowest EDC concentration category was selected to calculate the summary OR. The quality of the included studies was independently assessed by two investigators (DDY and YJ) using the Newcastle–Ottawa Scale [24]. This scale evaluates the selection of participants (four questions), the comparability (two questions), and the assessment of exposure/outcome (three questions). According to these parameters, the quality was divided into three grades increasing from low to high, where 0–4 points indicated low quality research, 5–6 points denoted moderate quality research, and 7–9 points represented high quality research. During the data extraction and quality assessment, any discrepancies were resolved by a joint reevaluation (JPY and HY) of the study.

### Data analysis

In the meta-analyses, the effect estimates were pooled if at least two original studies reported the same types of EDCs as follows: a) risk per unit increase in continuous exposure and b) risk of high versus low exposure level in the individual study. The OR and 95% CI were used as the primary measures to assess the relationship between EDCs and risk of GDM. The summary OR was calculated by using categorical exposure defined as high versus low because this method has been previously used in researches of environmental exposure studies [25]. Cochran *Q* and *I*^2^ statistics were used to evaluate the possible heterogeneity among the included studies, and *P* < 0.10 and *I*^*2*^ > 50% represent a significant level of heterogeneity [26, 27]. A fixed-effect model was performed when the overall summary OR revealed no obvious heterogeneity. Otherwise, a random-effect model was used. Publication bias among the included studies was assessed with Egger test and Begg tests [28, 29]. Sensitivity analyses were conducted to assess the sensitivity of our results. The statistical analyses were performed using Stata software (version 12.0), and the statistical significance was determined when *P* < 0.05 (two-sided).

## Results

### Search results and study characteristics

**Fig. 1:**
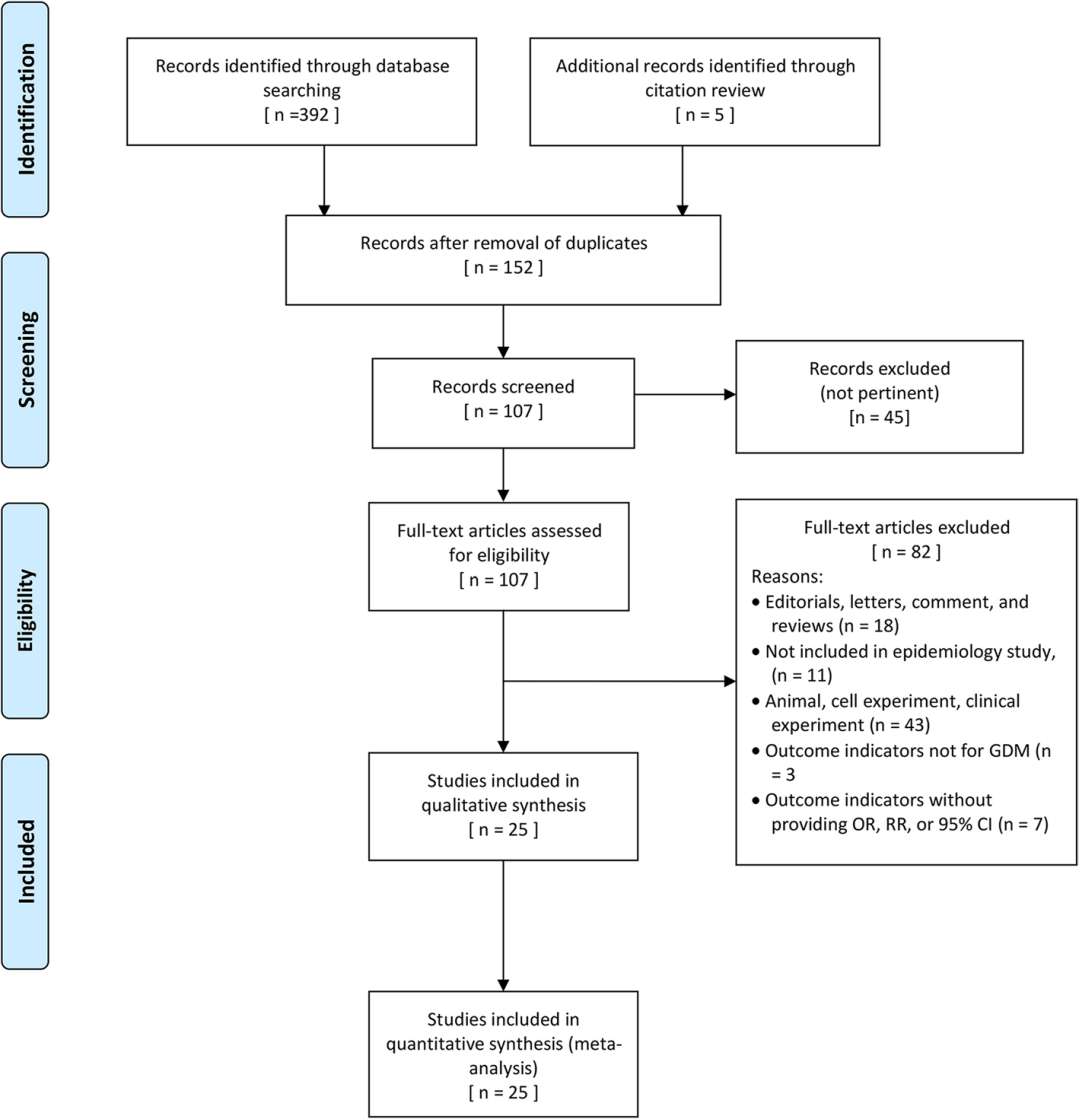
Flowchart of assessment of eligible studies

Approximately 392 articles were identified from three electronic databases using the search strategy, and five additional records were identified through citation review. Among those 397 literatures, 152 of which were left after removing duplicates. After screening the titles and abstracts, 45 articles were excluded for the following reasons: did not meet the selection criteria, irrelevant exposure or outcome, or studies not related to systematic review. According to the full-text article review, 25 studies were enrolled with a total of sample size of 23,796. The screening flow chart is displayed in Fig. 1.

Among those included articles, 16 were cohort studies, seven were case–control studies, and two were cross-sectional studies. In terms of study location, 11 studies were conducted in China, 7 in the USA, 2 in Canada, 2 in Spain, 1 in Iran, 1 in Norway, and 1 in the UK. More than half of those studies assessed the level of EDCs in serum (15 articles), six studies evaluated EDCs in urine, three studies detected EDCs in plasma, and one study assessed EDCs in meconium. In terms of laboratory technique, liquid chromatography was used in 17 studies and gas chromatography was used in eight studies. The general characteristics of the selected articles are displayed in Table 1. The assessment scores of the selected studies are in the range of 7–9 points, yielding an average of 7.9 score, suggesting a relatively high quality for all included articles. The results are shown in Additional file 3.

**Table 1:** Characteristics of included studies investigating the association between environmental endocrine disruptors and gestational diabetes mellitus

References (time period)	Location	Design	Used sample	Method	Sample size	Adjusting variables	Diagnostic criteria for GDM	Comparison categories	Endocrine disrupter	OR and 95%CI
Zhang et al. 2015 [30] (2005–2009)	China	Cohort	Serum	HPLC–MS/MS	258	Age (y), BMI (kg/m 2), parity conditional on gravidity (never pregnant/ pregnant without live birth/pregnant with previous birth), race/ethnicity (white/ nonwhite) and smoking (yes/no)	Self-report	Per SD increment	PFOS	1.13 (0.75–1.72)
									PFNA	1.06 (0.70–1.60)
									PFDA	1.04 (0.70–1.53)
									PFOA	1.86 (1.14–3.02)
Eslami et al. 2016 [31] (2013.09–2015.08)	Iran	Case–control	Serum	GC/MS	140	Maternal age (yrs), pre-pregnancy BMI (kg/m 2), gestational age (wks), and total lipids in maternal serum (mg/dL)	GDM diagnoses were made when two or more of the fast plasma glucose ≥ 92 mg/dL, ≥ 180 mg/dL at 1 h-OGTT or ≥ 153 mg/dL at 2 h-OGTT	Per unit increase in ln	PBDEs	2.21 (1.48–3.30)
									PBDE-99	2.14 (1.99–3.83)
									PBDE-28	2.73 (1.22–6.11)
									PCBs	1.75 (1.35–2.27)
									PCB-118	8.13 (2.78–23.73)
									PCB-153	2.41 (1.21–4.81)
									PCB-28	0.31 (0.14–0.67)
Vafeidi et al. 2017 [32] (2007.02–2008.02)	Spain	Cohort	Serum	GC–MS/MS	939	Gestational age at sampling (weeks), maternal age (b30 years, 30 years), pre-pregnancy BMI (kilograms per meter squared), parity (primiparous, multiparous), ma- ternal educational level [low ( 9 years of mandatory schooling), medium (N9 years of schooling up to attending postsecondary school education), high (attending university or having a university/technical college degree)], smoking during pregnancy (never, ever), gestational weight gain (kilograms) and maternal serum triglycerides and cholesterol	GDM diagnoses were made when two or more of the fast plasma glucose ≥ 95 mg/dL, ≥ 180 mg/dL at 1 h-OGTT, ≥ 155 mg/dL at 2 h-OGTT, or ≥ 140 mg/dL at 3 h-OGTT	Per tenfold increase	PCBs	4.56 (1.02–20.36)
Wang et al. 2018a [33] (2013.01–2013.03)	China	Case–control	Serum	UPLC	252	BMI, gestational weight gain, ethnic groups, maternal education, parity, maternal drinking during pregnancy, and household income	GDM diagnoses were made when the fast plasma glucose ≥ 95 mg/dL, ≥ 180 mg/dL at 1 h-OGTT, ≥ 155 mg/dL at 2 h-OGTT, or ≥ 140 mg/dL at 3 h-OGTT	Per 1 ng/mL increase in serum PFASs	PFOA	1.31 (0.95–1.80)
									PFNA	1.25 (0.37–4.28)
									PFDA	0.85 (0.30–2.92)
									PFOS	0.96 (0.85–1.09)
									PFUnDA	1.79 (0.65–4.96)
									PFHxS	1.07 (0.86–1.35)
Liu et al. 2018 [34] (2013.08–2015.06)	China	Case–control	Serum	GC-HRMS	439	Pregnancy BMI, serum triglyceride and total cholesterol	GDM was defined if a woman had any of the following plasma glucose values: (1) Fasting: ≥ 5.1 mmol/L; (2) 1 h: ≥ 10.0 mmol/L; and (3) 2 h: ≥ 8.5 mmol/L in the 75-g oral glucose tolerance test (OGTT)	Q4 vs. Q1	PBDE-28	2.39 (1.03–5.57)
									PBDE-47	2.01 (0.88–4.60)
									PBDE-99	2.01 (0.88–4.58)
									PBDE-100	2.04 (0.89–4.70)
									PBDE-153	3.42 (1.49–7.89)
									PBDE-154	1.70 (0.73–3.99)
									PBDEs	2.23 (1.04–5.00)
Zhang et al. 2018 [35] (2013.08–2015.06)	China	Case–control	Serum	GC-HRMS	231	no	GDM diagnoses were made when the fast plasma glucose ≥ 5.1 mmol/L, ≥ 10 mmol/L at 1 h-OGTT, ≥ 8.5 mmol/L at 2 h-OGTT	Na	PCB-28	1.86(1.05–3.27)
									PCB-52	1.90(1.28–2.82)
									PCB-101	1.85(1.22–2.82)
									PCB-138	1.51(0.90–2.53)
									PCB-153	1.45(0.88–1.88)
									PCB-180	1.25(0.83–1.88)
									PCBs	4.70(1.02–21.70)
Shapiro et al. 2015 [19] (2008–2010)	Canada	Cohort	Urine	LC–MS/MS	1274	Maternal age, race, pre-pregnancy BMI, education and specific gravity	GDM was defined if ≥ 2 of the following plasma glucose values: (1) Fasting: ≥ 5.3 mmol/L; (2) 1 h: ≥ 10.6 mmol/L; (3) 2 h: ≥ 9.2 mmol/L in the 75-g oral glucose tolerance test (OGTT)	Q4 vs. Q1	MEP	0.50 (0.20–1.40)
									MBP	0.60 (0.10–2.20)
									MBzP	1.50 (0.50–4.70)
									MCPP	0.60 (0.20–1.90)
									DEHP	0.90 (0.30–2.90)
Shapiro et al. 2016 [36] (2008–2010)	Canada	Cohort	Urine	GC/MSMS and UPLC-MS–MS	1274	Maternal age, race, pre-pregnancy BMI and education; analyses for organophosphorus pesticide metabolites are additionally adjusted for urinary specific gravity; analyses for PCBs and organochlorine pesticides are additionally adjusted for total lipids	GDM was defined if ≥ 2 of the following plasma glucose values: (1) Fasting: ≥ 5.3 mmol/L; (2) 1 h: ≥ 10.6 mmol/L; (3) 2 h: ≥ 9.2 mmol/L in the 75-g oral glucose tolerance test (OGTT)	Q4 vs. Q1	PFOA	0.90 (0.30–2.30)
									PFOS	0.70 (0.30–1.70)
									PFHxS	1.20 (0.40–3.50)
									PCB-118	1.40 (0.50–3.50)
									PCB-138	1.50 (0.50–4.20)
									PCB-153	1.40 (0.50–4.10)
									PCB-180	1.30 (0.50–3.50)
									PCBs	1.00 (0.30–2.70)
Jaacks et al. 2016 [37] (2005–2007)	USA	Cohort	Serum	HPLC–MS/MS	258	Total serum lipids estimated, age, and waist-to-height ratio, all specified continuously	GDM was identified from medical record	Na	PCBs	0.68 (0.31–1.49)
									PCB-28	0.90 (0.24–3.31)
									PCB-101	1.00 (0.69–1.47)
									PCB-118	0.81 (0.51–1.29)
									PCB-138	0.53 (0.29–0.99)
									PCB-153	0.48 (0.24–0.98)
									PCB-180	0.41 (0.19–0.87)
Smarr et al. 2016 [38] (2005–2009)	USA	Cohort	Serum	GC/MS	258	Serum lipids, age, BMI, non-white race, smoking, and the sum of remaining chemicals in the relevant class of compounds	GDM was identified from medical record	Na	PBDE-28	0.47 (0.17–1.26)
									PBDE-47	0.32 (0.10–1.01)
									PBDE-99	0.44 (0.15–1.33)
									PBDE-100	2.22 (0.96–5.17)
									PBDE-153	1.79 (1.18–2.74)
									PBDE-154	1.04 (0.34–3.17)
Rahman et al. 2019 [39] (2009.07–2013.01)	USA	Cohort	Plasma	UPLC	2334	Maternal age (continuous), enrollment BMI (19–24.9; 25–29.9), education (< college; some college/undergraduate; graduate/post graduate), parity (nulliparous; multiparous), race/ethnicity (white, African American, Hispanic, Asian), family history of type 2 diabetes among first degree relatives, serum cotinine level (continuous), and serum total lipids (continuous, mg/dL)	GDM was defined if fasting plasma glucose (FPG) ≥ 5.3 mmol/L, or 1-h plasma glucose (1 h-PG) ≥ 10.0 mmol/L, or 2-h plasma glucose (2 h-PG) ≥ 8.6 mmol/L, or 3-h plasma glucose ≥ 7.8 mmol/L	Per SD increment	PCB-101	1.03 (0.75–1.41)
									PCB-118	0.98 (0.71–1.36)
									PCB-138	0.99 (0.72–1.34)
									PCB-153	1.01 (0.77–1.32)
									PCB-180	1.08 (0.83–1.39)
									PCB-28	1.06 (0.89–1.27)
									PCB-52	1.13 (0.74–1.71)
									PCBs	0.99 (0.73–1.35)
									PFDA	0.72 (0.39–1.32)
									PFHxS	0.87 (0.52–1.46)
									PFNA	0.80 (0.50–1.27)
									PFOA	0.70 (0.43–1.14)
									PFOS	0.86 (0.60–1.23)
									PFUnDA	0.66 (0.37–1.19)
									PBDE-28	1.08 (0.94–1.23)
									PBDE-100	0.90 (0.49–1.66)
									PBDE-153	0.64 (0.26–1.57)
									PBDE-154	1.23 (1.12–1.34)
									PBDE-47	1.18 (1.08–1.29)
									PBDE-99	1.04 (0.92–1.15)
Matilla-Santander et al. 2017 [40] (2003–2008)	Spain	Cohort	Serum	HPLC–MS/MS	1240	Subcohort, country of birth, prepregnancy body mass index, previous breastfeeding, parity, gestational week at blood extraction, physical activity, and relative Mediterranean Diet Score	Results of the OGTT are routinely used to classify women as having GDM if two or more of the baseline or postchallenge blood glucose concentrations exceed National Diabetes Data Group (NDDG) reference values	Per tenfold increase	PFOA	1.20 (0.62–2.30)
									PFOS	2.40 (0.93–6.18)
									PFHxS	1.58 (0.73–3.44)
									PFNA	0.85 (0.40–1.80)
Valvi et al. 2017 [41] (1997–2000)	Norway	Cohort	Serum	HPLC–MS/MS	604	Maternal age at delivery, education, parity, pre-pregnancy BMI (continuous) and smoking during pregnancy	GDM was identified from medical record	Per unit increase	PCBs	0.97 (0.71–1.33)
									PFOS	0.86 (0.43–1.70)
									PFOA	0.79 (0.44–1.41)
									PFHxS	1.03 (0.80–1.33)
									PFDA	1.20 (0.73–1.96)
									PFNA	0.88 (0.53–1.47)
Neblett et al. 2020 [42] (2012–2015)	USA	Cross-sectional	Serum	GC/MS	254	Age (current age & age at pregnancy), BMI, and total lipid levels	Self-report	Na	PCBs	1.06 (0.59–1.87)
Fisher et al. 2018 [43] (2001–2009)	UK	Case–control	Serum	LC–MS	232	Age, pre-pregnancy body mass index (log-transformed), IMD (log-transformed), and parity	GDM was diagnosed if they meet one or more of the following criteria: Fasting plasma glucose ≥ 5.1 mmol/l, 60-min plasma glucose ≥ 10.0 mmol/l, or 120-min plasma glucose ≥ 8.5 mmol/l	Q4 vs. Q1	MEP	1.19 (0.42–3.37)
									MIBP	4.89 (1.32–18.14)
									MBP	1.42 (0.52–3.88)
Shaffer et al. 2019 [21] (2010–2012)	USA	Cohort	Urine	HPLC–MS/MS		Na	GDM is diagnosed in women with two or more abnormal values in the OGTT: fasting: 95 mg/dL; 1 h: 180 mg/dL; 2 h: 155 mg/dL; and 3 h: 140 mg/dL	Per interquartile-range increase	MEP	1.61 (1.10–2.36)
									MBP	1.13 (0.80–1.55)
									MCPP	1.06 (0.70–1.55)
									MBzP	1.03 (0.67–1.52)
									DEHP	1.05 (0.71–1.44)
									MEHP	1.03 (0.38–2.79)
									MIBP	1.15 (0.80–1.60)
Zhang et al. 2017 [44] (2013.05–2014.09)	China	Cohort	Urine	HPLC–MS/MS	3009	The FPG level of early stages pregnancy, maternal age, pre-pregnancy BMI, monthly household income, reproductive history, gestational weeks, concentration of urinary creatinine	GDM was diagnosed if fasting plasma glucose (FPG) ≥ 5.1 mmol/L (≥ 92 mg/dL), or 1 h plasma glucose (1 h-PG) ≥ 10.0 mmol/L (≥ 180 mg/dL), or 2 h plasma glucose (2 h-PG) ≥ 8.5 mmol/L (≥ 153 mg/dL)	Na	MEP	1.12 (0.83–1.52)
									MBP	1.49 (1.09–2.04)
									MBzP	0.93 (0.69–1.25)
Xu et al. 2020 [45] (2017.01–2019.01)	China	Case–control	Serum	UPLC-Q/TOF MS	1575	Maternal age, sampling time, parity, BMI, educational level, and serum lipids	GDM was diagnosed if fasting plasma glucose (FPG) ≥ 5.1 mmol/L, or 1 h plasma glucose (1 h-PG) ≥ 10.0 mmol/L, or 2 h plasma glucose (2 h-PG) ≥ 8.5 mmol/L	Per tenfold increase	PFOA	1.51 (0.63–3.84)
									PFOS	0.61 (0.42–1.65)
									PFDA	0.81 (0.21–2.01)
									PFNA	1.11 (0.49–2.85)
									PFHxS	1.09 (0.49–3.01)
									PFBS	1.69 (1.20–2.01)
									PFDoA	2.49 (1.07–3.72)
Preston et al. 2020 [46] (1999–2002)	USA	Cohort	Plasma	HPLC–MS/MS	1540	Maternal age, pre-pregnancy BMI, prior history of GDM, parity, race, ethnicity, smoking, education	GDM is diagnosed in women with two or more abnormal values in the OGTT: fasting: 95 mg/dL; 1 h: 180 mg/dL; 2 h: 155 mg/dL; and 3 h: 140 mg/dL	Q4 vs. Q1	PFOA	1.40 (0.70–2.90)
									PFOS	1.50 (0.70–3.00)
									PFNA	1.00 (0.50–2.00)
									PFHxS	1.00 (0.50–2.20)
Wang et al. 2018b [47] (2013.09–2014.12)	China	Cohort	Serum	UPLC-Q/TOF MS	560	Pregnant age, diabetes mellitus history of relatives, husband smoking status, family per capita income, baby sex, averaged intake of meat, vegetable,and aquatic products, averaged physical activity, averaged energy intake and pre-pregnant maternal BMI	GDM was diagnosed if fasting plasma glucose (FPG) ≥ 5.1 mmol/L, or 1 h plasma glucose (1 h-PG) ≥ 10.0 mmol/L, or 2 h plasma glucose (2 h-PG) ≥ 8.5 mmol/L	Q3 vs. Q1	PFOS	2.11 (0.76–5.86)
									PFOA	0.71 (0.29–1.75)
Gao et al. 2021 [48] (2013.05–2019.05)	China	Cohort	Urine	LC–MS	3273	Maternal age, pre-pregnancy BMI, income and primiparous	GDM was diagnosed if fasting plasma glucose (FPG) ≥ 5.1 mmol/L (≥ 92 mg/dL), or 1 h plasma glucose (1 h-PG) ≥ 10.0 mmol/L (≥ 180 mg/dL), or 2 h plasma glucose (2 h-PG) ≥ 8.5 mmol/L (≥ 153 mg/dL)	Per tenfold increase	MEP	1.18(0.96–1.46)
									MBP	1.20(0.95–1.53)
									MBzP	0.99(0.86–1.15)
									MEHP	0.96 (0.77–1.20)
									DEHP	0.95(0.68–1.33)
Guo et al. 2020 [49] (2013.07–2014.07)	China	Cross-sectional	Meconium	LC–MS/MS	251	Mother’s age, pre-pregnancy BMI and gestational age	GDM was diagnosed if fasting plasma glucose (FPG) ≥ 5.1 mmol/L (≥ 92 mg/dL), or 1 h plasma glucose (1 h-PG) ≥ 10.0 mmol/L (≥ 180 mg/dL), or 2 h plasma glucose (2 h-PG) ≥ 8.5 mmol/L (≥ 153 mg/dL)	Per unit increase in ln	MEP	1.40 (0.39–4.95)
									MBP	3.10 (0.87–11.21)
									MIBP	2.34 (1.01–5.43)
									MEHP	3.51 (1.24–9.92)
Zukin et al. 2021 [50] (1999–2000)	USA	Cohort	Urine		415	Maternal age, income, maternal education, marital status, sugar-sweetened beverage consumption, country of birth, and maternal pre-pregnancy BMI	GDM was diagnosis if either (1) maternal plasma glucose levels on the OGTT exceeded at least two of the following plasma levels: fasting 95 mg/dL (5.3 mmol/L), 1 h 180 mg/dL (10.0 mmol/L); 2 h 155 mg/dL (8.6 mmol/L); 3 h 140 mg/dL (7.8 mmol/L) or (2) a diagnosis of GDM in the maternal medical records	Na	MEP	1.10 (0.90–1.40)
									MBP	1.00 (0.80–1.50)
									MIBP	1.10 (0.80–1.40)
									MBzP	1.10 (0.80–1.50)
									DEHP	1.20 (0.80–1.70)
									MCPP	1.00 (0.70–1.40)
Liu et al. 2019 [51] (2013.08–2015.06)	China	Case–control	Serum	GC-HRMS	439	Maternal age, pregnancy BMI, fetal sex, and serum triglyceride and total cholesterol	GDM was defined if a woman had any of the following plasma glucose values: (1) Fasting: ≥ 5.1 mmol/L; (2) 1 h: ≥ 10.0 mmol/L; and (3) 2 h: ≥ 8.5 mmol/L in the 75-g oral glucose tolerance test (OGTT)	Per unit increase in ln	PFOS	1.36 (0.88–2.11)
									PFOA	1.23 (0.92–1.64)
Yu et al. 2021 [52] (2013–2016)	China	Cohort	Plasma	HPLC–MS/MS	2747	Maternal age, pre-pregnant BMI, maternal education, smoking status, parity, averaged physical activity and economic status	GDM was diagnosed if fasting plasma glucose (FPG) ≥ 5.1 mmol/L, or 1 h plasma glucose (1 h-PG) ≥ 10.0 mmol/L, or 2 h plasma glucose (2 h-PG) ≥ 8.5 mmol/L	Per tenfold increase	PFOA	1.11 (0.83–1.15)
									PFOS	1.10 (0.88–1.36)
									PFNA	1.03 (0.81–1.30)
									PFDA	0.95 (0.78–1.16)
									PFHxS	1.15 (0.86–1.54)
									PFUnDA	0.91 (0.74–1.12)
									PFDoA	0.99 (0.78–1.26)
									PFBS	1.23 (1.05–1.44)

Abbreviations: *SD* standard deviation, *BMI* body mass index, *FPG* fasting plasma glucose,*1 h-PG* 1 h plasma glucose, *2 h-PG* 2 h plasma glucose, *GDM* gestational diabetes mellitus, *OGTT* oral glucose tolerance test, *GC–MS* gas chromatography coupled to mass detection, *GC/MS* gas chromatography mass spectrometry, *LC–MS* liquid chromatography coupled to mass spectrometry, *GC–MS/MS* gas chromatograph triple quadrupole mass spectrometer, *UPLC-MS/MS* ultra-performance liquid chromatography coupled with triple quadrupole tandem mass spectrometry, *HPLC* high performance liquid chromatography, *GC-HRMS* gas chromatography-high resolution mass spectrometry, *LC–MS/MS* liquid chromatography coupled with triple quadrupole tandem mass spectrometry, *UPLC-Q/TOF MS* ultra-performance liquid chromatography coupled to quadrupole time-of-flight mass spectrometry, *T2D* type 2 diabetes, *ppBMI* pre-pregnancy BMI, *PBDEs* Polybrominated diphenylethers, *PCBs* Polychlorinated biphenyls, *PAEs* phthalates, *PFAS* Per-and polyfluoroalkyl substances, *PFNA* Perfluorononanoic acid, *PFOA* Perfluorooctanoic acid, *PFDA* Perfluorodecanoic acid, *PFHxS* Perfluorohexanesulfonic acid, *PFOS* Perfluorooctanesulfonic acid, *PFUnDA* Perfluoroundecanoic acid, *PFDoA* perfluorododecanoic acid, *PFBS* perfluorobutane sulfonate, *EtFOSAA* 2-(N- ethyl-perfluorooctane sulfonamide) acetate, *MeFOSAA* 2-(N-methyl-perfluorooctane sulfonamide) acetate, *DEHP* diethylhexyl phthalate, *MBP* mono-n-butyl phthalate, *MBzP* mono-benzyl phthalate, *MCPP* mono-3-carboxypropyl phthalate, *MEP* mono-ethyl phthalate, *MIBP* mono-isobutyl phthalate, *MEHP* mono-(2-ethylhexyl) phthalate

According to the items in the inclusion criteria (the summary effect estimates were calculated if the endocrine-disrupting chemical exposure have been examined in ≥ 2 studies as follows: a) risk per unit increase in continuous exposure and b) risk of high versus low exposure level in the individual study) and the searching results, the EDCs evaluated in this work are: PBDEs, PCBs, PAEs, PFAS, perfluorononanoic acid (PFNA), perfluorooctanoic acid (PFOA), perfluorodecanoic acid (PFDA), perfluorohexanesulfonic acid (PFHxS), perfluorooctanesulfonic acid (PFOS), perfluoroundecanoic acid (PFUnDA), perfluorododecanoic acid (PFDoA), perfluorobutane sulfonate (PFBS), 2-(N- ethyl-perfluorooctane sulfonamide) acetate (EtFOSAA), 2-(N-methyl-perfluorooctane sulfonamide) acetate (MeFOSAA), diethylhexyl phthalate (DEHP), mono-n-butyl phthalate(MBP), mono-benzyl phthalate(MBzP), mono-3-carboxypropyl phthalate (MCPP), mono-ethyl phthalate (MEP), mono-isobutyl phthalate (MIBP), and mono-(2-ethylhexyl) phthalate (MEHP).

### Polychlorinated biphenyls and the risk of GDM

**Fig. 2:**
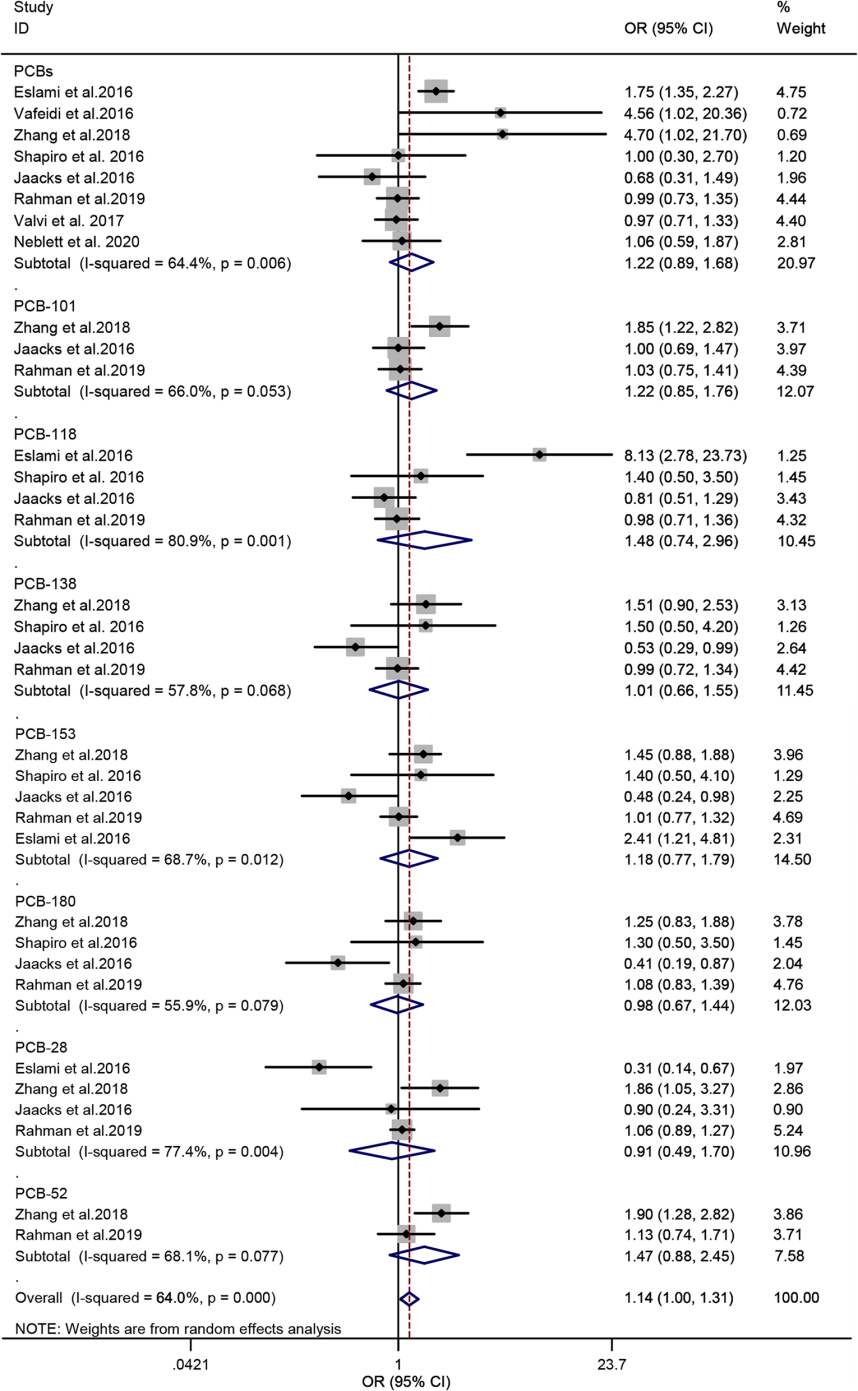
Forest plot of PCBs exposure and risk of GDM. The points represent the study- specific odds ratios (ORs) and the horizontal lines correspond to 95% confidence intervals (CIs). The study-specific weight is presented as the grey areas. The pooled ORs and 95% CIs are presented as the diamonds. The vertical dashed line represents an OR of 1.14

Eight studies [31, 32, 35–37, 39, 41, 42] were included in the analysis of PCBs and the risk of GDM. The PCBs, namely, PCB-101, PCB-118, PCB-138, PCB-153, PCB-180, PCB-28, and PCB-52, and total PCBs were enrolled in this study. The result of the heterogeneity test indicated significant heterogeneity among the included studies (*P* = 0.000, *I*^*2*^ = 64.0%). Accordingly, a random-effect model was used, and the overall estimated OR exhibited a significant association between PCBs and the risk of GDM (OR = 1.14; 95% CI = 1.00–1.31, *n* = 8), indicating that PCBs is a risk factor for GDM. The forest plot is displayed in Fig. 2. In the subgroup analysis, no significant association was observed between the eight PCB congeners and GDM risk. When the subgroup analysis was classified by the design of studies, the summary OR from the cohort and non-cohort studies were 0.99 (95% CI = 0.91–1.09; *n* = 5) and 1.60 (95% CI = 1.23–2.09; *n* = 3), respectively (Table 2).

**Table 2:** Endocrine-disrupting chemicals and the risk of gestational diabetes mellitus: the summary ORs of cohort and non-cohort studies and the results of publication bias

ECDs	Type	No. of studies	Effect size, pooled OR (95% CI)	Heterogeneity		Publication bias	
				*P* value	*I*^*2*^ value, %	Egger *P* value	Begg *P* value
PCBs	Cohort	5	0.99 (0.91–1.09)	0.394	5.0	0.539	0.933
	Non-cohort	3	1.60 (1.23–2.09)	0.000	68.2	0.790	0.631
PBDEs	Cohort	2	1.12 (1.00–1.26)	0.006	57.8	0.221	0.15
	Non-cohort	2	2.21 (1.83–2.68)	0.993	0.0	0.639	0.721
PAEs	Cohort	5	1.08 (1.02–1.15)	0.391	0.0	0.666	0.597
	Non-cohort	2	2.17 (1.45–3.26)	0.353	0.0	0.609	0.368
PFASs	Cohort	8	1.06 (1.00–1.12)	0.574	0.0	0.848	0.395
	Non-cohort	3	1.22 (1.04–1.44)	0.010	59.1	0.263	0.621

Abbreviations: *PCBs* polychlorinated biphenyls, *PBDEs* poly-brominated diphenyl ethers, *PFAS* per- and polyfluoroalkyl substances, *PAEs* phthalates, *CI* confidence interval, *OR* odds ratio

### Polybrominated diphenyl ethers and risk of GDM

**Fig. 3:**
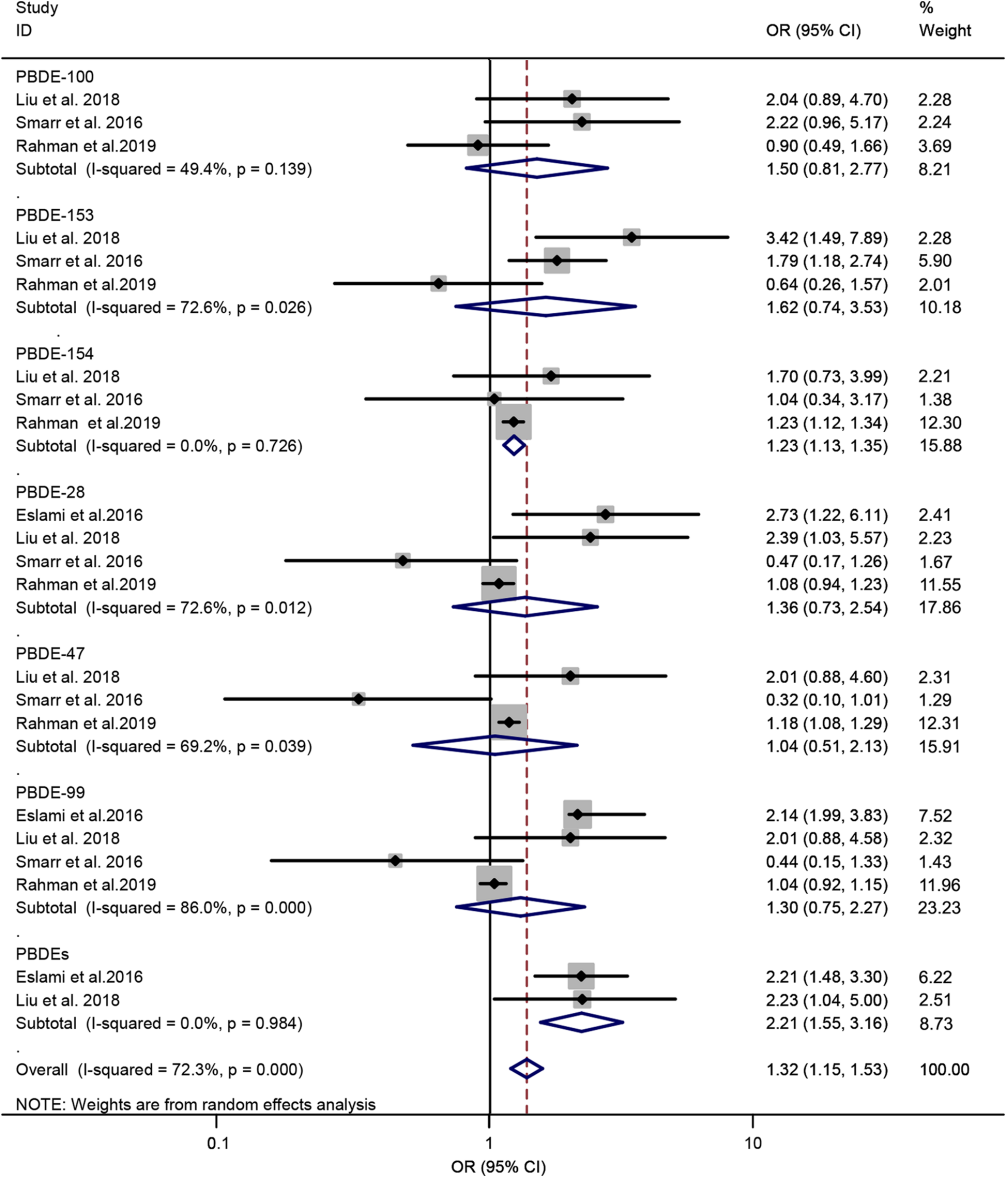
Forest plot of PBDEs exposure and risk of GDM. The points represent the study- specific odds ratios (ORs) and the horizontal lines correspond to 95% confidence intervals (CIs). The study-specific weight is presented as the grey areas. The pooled ORs and 95% CIs are presented as the diamonds. The vertical dashed line represents an OR of 1.32

Four literatures [31, 34, 38, 39] were enrolled to investigate the association between PBDEs and risk of GDM. The subgroup analysis was performed according to PBDE congeners. Six PBDE congeners, namely, PBDE-100, PBDE-153, PBDE-154, PBDE-28, PBDE-47, and PBDE-99, and total PBDEs were included. A random-effect model was used to estimate the summary OR, and the result displayed a remarkable correlation between PBDEs and risk of GDM (OR = 1.32; 95% CI = 1.15–1.53; *n* = 4), with a significant heterogeneity among the included studies (*P* = 0.00, *I*^*2*^ = 72.3%). The forest plot is displayed in Fig. 3. The subgroup analysis displayed that PBDE-154 significantly contributed to the risk of GDM (OR = 1.23; 95% CI = 1.13–1.35), indicating that PBDE-154 may be a risk factor for GDM. Meanwhile, the pooled OR of the total PBDEs was 2.21 (95% CI = 1.55–3.16), suggesting that the total PBDEs is a risk factor for GDM. Meanwhile, the summary ORs of the other PBDE congeners (PBDE-100, PBDE-153, PBDE-28, PBDE-47, and PBDE-99) did not show a significant association. When the subgroup analysis was classified by the design of studies, the summary OR from the cohort and non-cohort studies were 1.12 (95% CI = 1.00–1.26; *n* = 2) and 2.21 (95% CI = 1.83–2.68; *n* = 2), respectively (Table 2).

### Phthalates and risk of GDM

**Fig. 4:**
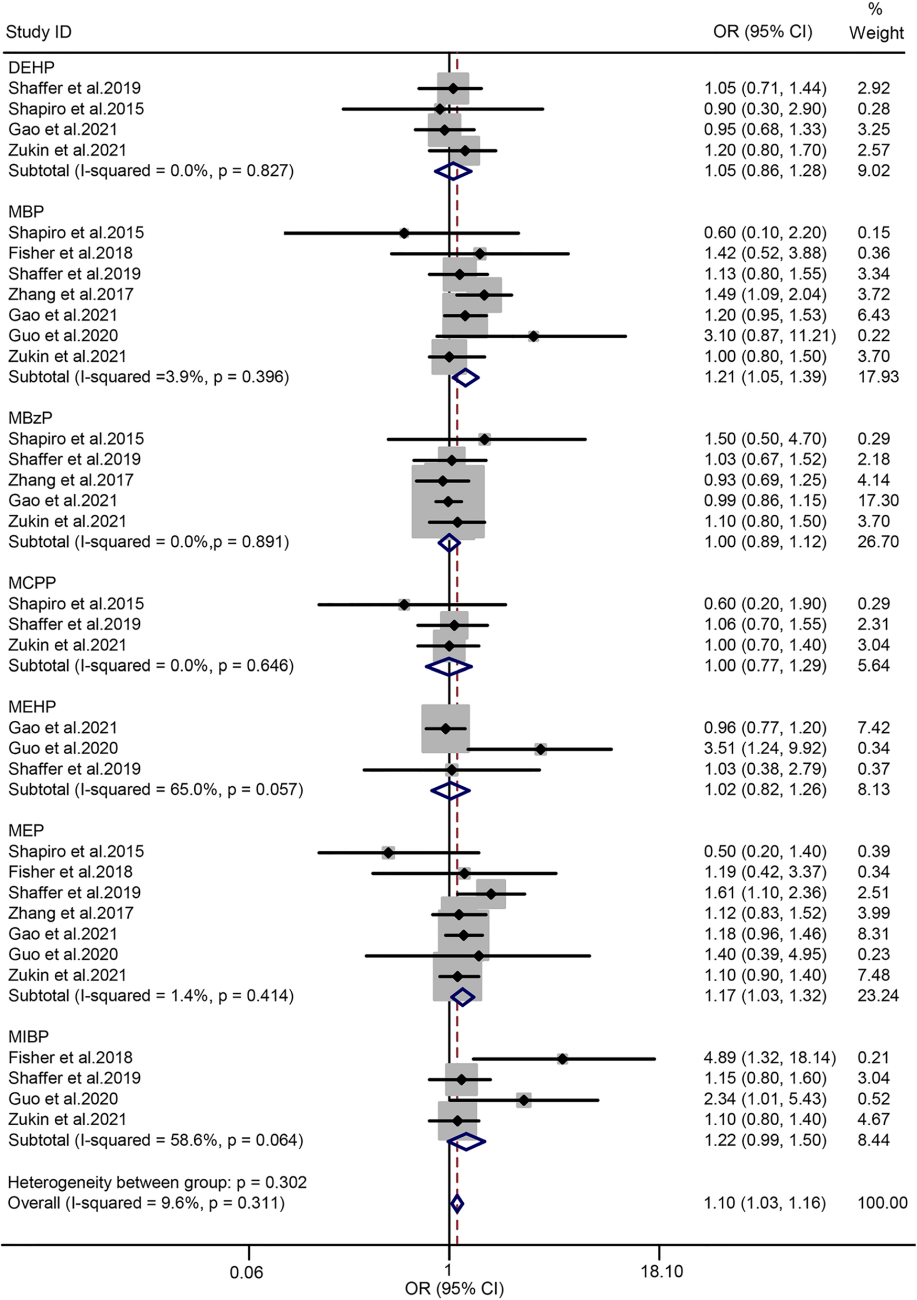
Forest plot of PAEs exposure and risk of GDM. The points represent the study- specific odds ratios (ORs) and the horizontal lines correspond to 95% confidence intervals (CIs). The study-specific weight is presented as the grey areas. The pooled ORs and 95% CIs are presented as the diamonds. The vertical dashed line represents an OR of 1.10

Seven studies [19, 21, 43, 44, 48–50] were selected to assess the association between PAEs and GDM, and the subgroup analysis was performed according to the PAE metabolites. Seven metabolites, namely, DEHP, MBP, MBzP, MCPP, MEHP, MEP, and MiBP were enrolled. The heterogeneity test proved that the *I*^*2*^ of the summary PAEs was 9.6% (*P* = 0.311). Accordingly, a fixed-effect model was used to combine data. The forest plot is shown in Fig. 4. The summary OR indicated a significantly positive association between PAEs and risk of GDM (OR = 1.10; 95% CI = 1.04–1.16; *n* = 7). In the subgroup analysis, MBP and MEP exposure were markedly associated with the risk of GDM (OR = 1.21; 95% CI = 1.05–1.39 for MBP; OR = 1.17; 95% CI = 1.03–1.32 for MBP). Meanwhile, the OR values of the other five PAE metabolites indicated no statistical significance. When the subgroup analysis was classified according to the design of studies, the summary OR from the cohort and non-cohort studies were 1.08 (95% CI = 1.02–1.15; *n* = 5) and 2.17 (95% CI = 1.45–3.26; *n* = 2), respectively (Table 2).

### Per- and polyfluoroalkyl substances and risk of GDM

**Fig. 5:**
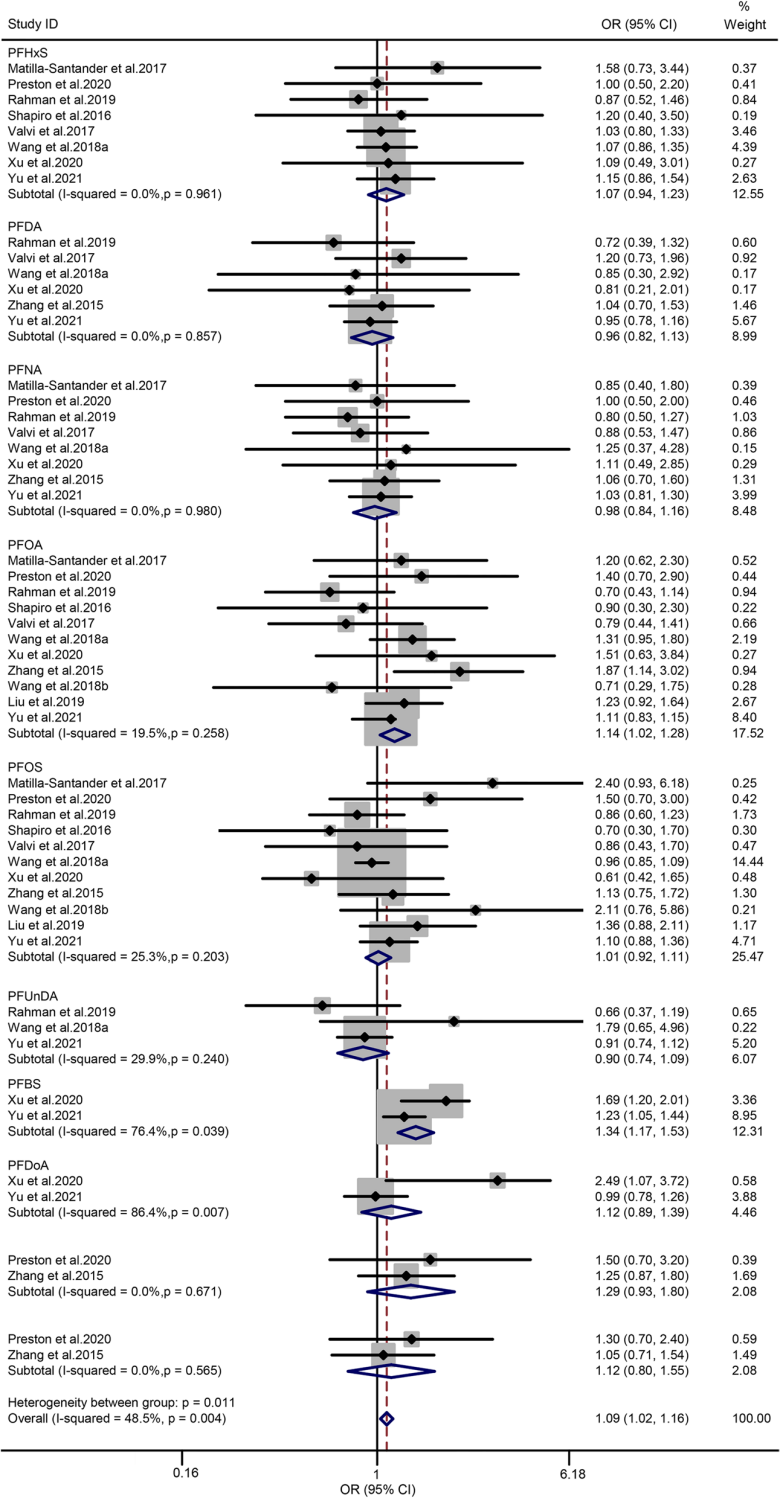
Forest plot of PFAS exposure and risk of GDM. The points represent the study- specific odds ratios (ORs) and the horizontal lines correspond to 95% confidence intervals (CIs). The study-specific weight is presented as the grey areas. The pooled ORs and 95% CIs are presented as the diamonds. The vertical dashed line represents an OR of 1.04

Eleven articles [30, 33, 36, 39–41, 45–47, 51, 52] were included in the analysis of PFAS and the risk of GDM. The PFAS congeners enrolled in this study included PFDA, PFHxS, PFNA, PFOA, PFOS, PFUnDA, PFBS, PFDoA, EtFOSAA, and MeFOSAA. Heterogeneity among the enrolled articles indicated no statistical significance (*P* = 0.011 and *I*^*2*^ = 48.5%), suggesting that the results of the included studies were statistically homogeneous. Thus, a fixed-effect model was used to evaluate the overall estimated OR and 95% CIs. The summary OR of PFAS and the risk of GDM was 1.09 (95% CI = 1.02–1.16; *n* = 11), indicating an additive effect on the risk of GDM. The forest plot is displayed in Fig. 5. In the subgroup analysis, the pooled OR value of the PFBS was markedly associated with the risk of GDM (OR = 1.37; 95% CI = 1.17–1.53). Meanwhile, the pooled OR values of the other nine PFAS congeners and risk of GDM exhibited no significant association. When the subgroup analysis was classified according to the design of studies, the summary OR from the cohort and non-cohort studies were 1.06 (95% CI = 1.00–1.12; *n* = 8) and 1.22 (95% CI = 1.04–1.44; *n* = 3), respectively (Table 2).

### Publication bias and sensitivity analyses

The Egger and Begg tests for PCBs provided no evidence of substantial publication bias (*P* = 0.305 for Egger’s test; *P* = 0.591 for Begg’s test), as shown in Table 3. Neither the Egger’s test nor the Begg’s test for PBDEs reached significance (*P* = 0.130 for Egger’s test; *P* = 0.195 for Begg’s test). The *P* values of Egger and Begg tests for PAEs were 0.069 and 0.198, respectively. These results were not statistically significant. Furthermore, the Egger and Begg tests for PFAS also provided no publication bias (*P* = 0.535 for Egger’s test; *P* = 0.416 for Begg’s test). Thus, no publication bias was observed in the included studies. Sensitivity analyses were evaluated by leave-one-out-method. We omitted each study from the analysis one by one. As shown in Additional Fig. 1, the results of PCBs, PBDEs, PAEs, and PFAS were either marginal significant or significant, indicating stability of that the results of the present meta-analysis.

**Table 3:** Endocrine-disrupting chemicals and the risk of gestational diabetes mellitus: Summary ORs and the results of publication bias

ECDs	No. of studies	Effect size, pooled OR (95% CI)	Heterogeneity		Publication bias	
			*P* value	*I*^2^value, %	Egger *P* value	Begg *P* value
PCBs	8	1.14 (1.00–1.31)	0.000	64.0	0.305	0.591
PBDEs	4	1.32 (1.15–1.53)	0.000	72.3	0.130	0.195
PAEs	7	1.10 (1.03–1.16)	0.302	9.6	0.069	0.198
PFAS	11	1.09 (1.02–1.16)	0.011	48.5	0.535	0.416

Abbreviations: *PCBs* polychlorinated biphenyls, *PBDEs* poly-brominated diphenyl ethers, *PFAS* per- and polyfluoroalkyl substances, *PAEs* phthalates, *CI* confidence interval, *OR* odds ratio

## Discussion

EDCs are a special type of chemicals capable of mimicking or interfering with the endocrine system, thereby altering some key biological processes, such as reproduction, immunity, and metabolism [14]. Humans are exposed to EDCs via air inhalation, drinking water, and dermal absorption due to their extensive and worldwide use [53]. Several EDCs including PCBs, PBDEs, and PAEs have been shown to increase the risk of T2DM via different mechanisms [54–56]. Pregnancy is a time when woman’s health is particularly susceptible. Exposure to EDCs is especially concerning during pregnancy because more than 50 different chemical combinations can be exposed [57]. Compensatory insulin secretion from the pancreatic β-cells resolves insulin resistance from placental hormone flooding during a healthy pregnancy. GDM occurs when the maternal pancreatic β-cells are dysfunctional and unable to balance the increased insulin demand [5]. Several in vivo and animal studies indicated that pancreatic β-cells were the target for some well-known EDCs [58–60], suggesting that EDCs-induced pancreatic β-cells damage may be one of the important mechanisms in GDM development. The relationship between EDC exposure and risk of GDM has attracted considerable attention, in recent years [14, 61]. Several researchers have proposed the assumption that exposure to EDCs is a potential risk factor for GDM [19, 36]. This hypothesis has been validated by several epidemiological studies. For instance, some previous studies indicated that exposure to PFAS and phthalates in adulthood might be associated with impaired glucose tolerance, obesity, and gestational diabetes [14, 62, 63]. In a previous LIFECODES pregnancy cohort study, researchers found that MEP exposure was associated with a significant increase in impaired glucose tolerance (OR = 7.18, 95% CI: 1.97 –26.15) in the second trimester, whereas the concentration DEHP was inversely related to impaired glucose tolerance (OR = 0.25, 95% CI: 0.08 – 0.85) [20]. In one of our selected cohort studies, the authors found that urinary concentration of MEP was positively associated with the risk of GDM (OR = 1.61, 95% CI: 1.10 – 2.36), whereas the urinary concentration of MCPP was negatively correlated with the odd of GDM (OR = 0.64, 95% CI: 0.43 – 0.96) [21]. The results of these epidemiological studies are controversial, and meta-analysis is an important approach that can be used to reveal trends that may not be apparent in a single epidemiological research. Therefore, a comprehensive systematic review is necessary to assess the relationship between EDC exposure and GDM risk. However, relevant meta-analyses have not been performed until now due to the limited number of original studies, and little is known about the mechanisms underlying EDC exposure and GDM risk.

To our knowledge, this study is the first comprehensive meta-analysis research that created group modes according to its congeners to assess the possible association between EDCs and GDM. In this systematic review and meta-analysis, we explored the relationship between EDCs (including PCBs, PBDEs, PAEs, and PFAS) and risk of GDM. The data indicated that certain types of EDC exposure, including PCBs, PBDEs, PAEs, and PFASs, are risk factors for GDM. Our results suggest that exposure to certain types of EDCs are one of the modifying risk factors for GDM.

PCBs belong to the persistent organic pollutants, which can bioaccumulate and biomagnify within the food chain. PCB congeners are frequently detected in human milk and serum due to their persistent characteristics. Previous epidemiology studies have indicated that PCBs differ in their effects on obesity, insulin resistance, or diabetes depending on the number of chlorine atoms; heavily chlorinated PCBs were more likely to be associated with these effects; however, such patterns were not consistent among studies [39, 64, 65]. Individuals are exposed to various complex mixtures. These complex exposure models may produce synergistic, additive, or antagonistic effects on the human health. In our present study, the synergistic effect of multiple PCBs congeners demonstrated a significant influence on the prevalence of GDM even though single PCB congeners presented null associations in subgroup analysis. Currently, there are limited experimental data exploring the risk of GDM with prenatal PCB exposure. A previous study indicated that the activation of peroxisome proliferator-activated receptor alpha (PPARα) via interacting with the aryl hydrocarbon receptor is a potential biological mechanism of the association between PCBs and diabetes [66]. Another research using animal subjects demonstrated that sub-chronic exposure to PCBs disrupted the glucose homeostasis, suppressed the functions of pancreatic β-cells and reduced the insulin levels in mice [54]. Although these mechanisms were not necessarily generalizable to GDM due to the significant physiological changes that occur during pregnancy, these results still provide relevant evidence to some extent. Therefore, PCB exposure might have a significant influence on the prevalence of GDM, but the underlying mechanisms need to be further investigated.

PBDEs are a class of persistent organic pollutants that are commonly used as flame retardants in household consumer products such as electronics and furniture. PBDEs can leach into the environment and enter the human body, and it is associated with the endocrine and numerous health problems [67]. The result of PBDEs in the present study was in line with the enrolled literatures, which support the notion that PBDEs exposure is a risk factor of GDM. Dysglycemia displayed as continuum hyperglycemia during pregnancy is the main pathophysiological change in GDM. Mechanistically, the main pathways of PBDEs leading to GDM may be multifaceted. A previous study that used animal subjects to explore the biological plausibility for an association between PBDEs and GDM found a dose–response hyperglycemia and significant oxidative damage after exposure to PBDE congeners for 8 weeks [56]. Analogously, according to an in vitro study, exposure to PBDE congeners (BDE-47 and BDE-85) can directly stimulate insulin secretion via activating a thyroid receptor and Akt signaling in pancreatic β-cells [68]. T2D and GDM may be two clinical features of the same entity. The pathogenesis of both conditions are attributed to the continuum dysglycemia with the development of insulin resistance or impaired insulin secretion [34]. These data suggested that there are similar biologic pathways explaining EDC exposure because it is associated with diabetes in the general and gravid populations. Although the precise underlying mechanisms between PBDE exposure and GDM have not been elucidated, our findings together with the biological evidence indicated that PBDEs can potentially contribute to the development of GDM.

PAEs are plasticizers used in many consumer applications, such as food packaging, personal care products, and medical devices. They can migrate into food and enter the human body. Several metabolites of PAEs can be detected in the urine of > 75% of the general population [69]. The result of PAEs in the present study showed that it is a risk factor of GDM, indicating that the metabolic process of PAEs in the body could impair glucose metabolism and induce continuum hyperglycemia. Currently, the main mechanisms of PAEs and their metabolites contributing to the development of GDM are multifaceted. First, PAEs are a type of endocrine disruptors associated with the alterations of steroid hormones; several PAEs have been found to be capable to binding with estrogen receptor α and exhibit estrogenic activity [70]. Considerable evidence suggests that changes in the estrogens level are associated with insulin resistance, alterations in adipocytes, and metabolic disorders in women [71–73]. Second, the prolonged activation of estrogen receptor ERα by the environmental estrogen could lead to excess insulin release, pancreatic β-cell exhaustion, and peripheral insulin resistance, which may result in glucose metabolism disorder and contribute to the development of diabetes [74]. Moreover, PAEs can selectively regulate PPARα and in turn affect lipid modulation and glucose homeostasis, resulting in insulin resistance, which also contributes to the development of diabetes [75]. The subgroup analysis of our present study showed that five single PAE metabolites presented no risk, except for MBP. However, the synergistic effect of the six PAE metabolites on GDM verified this viewpoint in the aforementioned mechanism studies. Thus, PAEs metabolites might contribute to the development of GDM, and further studies are needed to assess and determine the precise underlying mechanisms.

PFASs have been extensively used in a series of industrial applications, such as product surfactants, paper and textile coatings, nonstick frying pan coatings, and repellents, resulting in ubiquitous contamination and worldwide exposure [76]. It can biomagnify in the food chain and bioaccumulate in human tissues due to their persistent characteristic. PFASs are structurally homologous to fatty acids and possess endocrine-disrupting properties, which may be associated with the development of lipid alterations and energy metabolism dysfunction [77]. Currently, evidence on the relationship between PFAS exposures and risk of GDM remains uncertain. Data from animal studies indicated that PFAS exposure can inhibit the phosphatidylinositol 3-kinase- serine/ threonine protein kinase signaling, which may interfere with the metabolic actions of insulin [78]. PFASs have been proven to be related to β-cell function, glycated hemoglobin, fasting proinsulin, and insulin secretion in the general population [79]. In our present study, exposure to PFASs did have an additive effect on the risk of GDM. Human beings are generally exposed to a series of PFAS mixtures. The sufficiently wide range of exposure and a large sample size are critical in the assessment of the relationship between PFAS exposure and risk of GDM [33]. In our present study, the summary OR from the cohort and case–control studies were indicated an additive effect on the risk of GDM. Thus, PFAS metabolites might contribute to the development of GDM, and further studies are needed to assess and determine the underlying mechanisms.

At present, the biological mechanisms of the potential relationship between EDC exposure and risk of GDM are not fully elucidated. As previously mentioned, GDM may share certain similar pathways for development in common with T2DM in terms of EDC exposure because both of them are characterized by continuum hyperglycemia and insulin resistance. Women with GDM are more likely to acquire T2DM later in life. Therefore, certain hypothesized mechanisms, such as mitochondrial dysfunction, oxidative stress, inflammation response, and insulin resistance, which have been considered to be associated with the development of diabetes may also be applicable to GDM [80]. The highlights of the present study are as follows. Firstly, this is the first comprehensive meta-analysis research to explore the potential association between EDCs exposure and the risk of GDM. Secondly, the estimated scores of all the included studies are higher than or equal to 7, indicating a relatively high quality of the original studies. Thirdly, the results of the sensitivity analysis did not significantly modify the conclusions of the present study, and no publication bias was observed in the included studies. Moreover, the results of the present meta-analysis reveal trends that may not be apparent in a single epidemiological study, and the persuasiveness of the results can be enhanced with a large sample size.

However, several limitations should be recognized in our present study. First, the types of EDCs and complications of pregnancy in this study are limited due to the insufficient number of relevant literatures and the failure of data extraction,. Second, the dose–response relationship between the individual pollutants and the risk of GDM was not established in the present study because humans are generally exposed to a complex mixtures of pollutants. The effects of individual compounds are difficult to distinguish. Third, the publication bias may be inevitable, even though no publication bias was observed in the Egger and Begg tests, because the unpublished data were not retrieved. Moreover, we did not perform a subgroup analysis to assess the relationship between the EDCs and the risk of GDM in different gestational stages (various trimesters) due to the limited number of included studies. In addition, the summary overall effect sizes reported in our meta-analysis are relatively small, particularly for cohort studies, even though all of our four results showed significant impact on the incidence of GDM due to the limited number of existing epidemiological studies and sample size in a single study. Finally, we are unable to perform more additional subgroup analyses to explore the possible sources of heterogeneity among the included studies due to the limited number of included studies especially for the PBDEs and PCBs exposure. Thus the study heterogeneity may be inevitable.

## Conclusion

In conclusion, the results of our present meta-analysis indicated that PCB, PBDE, PAE, and PFAS exposure have significant effects on the risk of GDM. These results provide strong evidence to a certain extent, supporting the hypothesis that certain EDCs (especially the PCBs, PBDEs, PAEs, and PFASs) exposure is related to an increased risk of GDM. Further large-sample and high-quality epidemiologic studies with improved methods for documenting cases of GDM and in vivo and in vitro studies are needed to verify the potential relationship and biological mechanisms.

## Supplementary Information

**Additional file 1: **PRISMA Report (modified) for Systematic Reviews.

**Additional file 2: **Search strategy for electronic databases.

**Additional file 3: **Table 2. Quality assessment of the included studies.

**Additional file 4: **Datasets.

**Additional file 5: Figure 1. **funnel plot of PCBs (A), PBDEs(B), PAEs(C), and PFAS (D) exposure and risk of GDM.

## Data Availability

The datasets used and/or analyzed during the current study are available as Additional file 4.

## Abbreviations

GDM: Gestational diabetes mellitus
EDCs: Endocrine-disrupting chemicals
T2D: Type 2 diabetes
PBDEs: Polybrominated diphenylethers
PCBs: Polychlorinated biphenyls
PAEs: Phthalates
PFAS: Per-and polyfluoroalkyl substances
PFNA: Perfluorononanoic acid
PFOA: Perfluorooctanoic acid
PFDA: Perfluorodecanoic acid
PFHxS: Perfluorohexanesulfonic acid
PFOS: Perfluorooctanesulfonic acid
PFUnDA: Perfluoroundecanoic acid
PFDoA: Perfluorododecanoic acid
PFBS: Perfluorobutane sulfonate
EtFOSAA: 2-(N- ethyl-perfluorooctane sulfonamide) acetate
MeFOSAA: 2-(N-methyl-perfluorooctane sulfonamide) acetate
DEHP: Diethylhexyl phthalate
MBP: Mono-n-butyl phthalate
MBzP: Mono-benzyl phthalate
MCPP: Mono-3-carboxypropyl phthalate
MEP: Mono-ethyl phthalate
MIBP: Mono-isobutyl phthalate
MEHP: Mono-(2-ethylhexyl) phthalate
