# Supplementary material for: Endocrine-disrupting chemicals and the risk of gestational diabetes mellitus: a systematic review and meta-analysis
Source: Environ Health. 2022 May 16;21:53. doi: 10.1186/s12940-022-00858-8 (PMC9109392; doi:10.1186/s12940-022-00858-8)
Supplement: Supplementary file 2 — Additional file 2: Search strategy for electronic databases. [file 12940_2022_858_MOESM2_ESM.doc]

Endocrine-disrupting chemicals and the risk of gestational diabetes mellitus: A systematic review and meta-analysis

Dandan Yan^1^, Yang Jiao^2^, Honglin Yan^1^, Tian Liu^1^, Hong Yan^2^, Jingping Yuan^1^

^1^Department of Pathology, Renmin Hospital of Wuhan University, 238 Jiefang-Road, Wuchang District, Wuhan, 430060, P.R. China. ^2^Department of Health Toxicology, MOE Key Lab of Environment and Health, School of Public Health, Tongji Medical College, Huazhong University of Science and Technology, 13 Hangkong-Road, Wuhan, 430030, P.R. China.

***Corresponding authors**: Jingping Yuan, PhD

Department of Pathology, Renmin Hospital of Wuhan University, 238 Jiefang-Road, Wuchang District, Wuhan, 430060, PR China.

Email: yuanjingping@whu.edu.cn (JP. Yuan) Tel: +86-027-88041911-85523.

**Supplementary material**

Search strategy for electronic databases

The following electronic databases were searched PubMed, EMBASE, Web of Science Core Collection.

**1. Search strategy for MEDLINE (PubMed) 143**

#1 (Diabetes, Pregnancy-Induced) OR (Diabetes, Pregnancy Induced) OR (Pregnancy-Induced Diabetes) OR (Gestational Diabetes*) OR (Diabetes Mellitus, Gestational) OR (Gestational Diabetes Mellitus) OR GDM [MeSH]

#2 (polychlorinated biphenyls [MeSH]) OR (Biphenyls, Polychlorinated) OR (Polychlorobiphenyl Compounds) OR (Compounds, Polychlorobiphenyl) OR (Polychlorinated Biphenyl) OR (Biphenyl, Polychlorinated) OR PCBs

#3 (poly-brominated diphenyl ethers [MeSH]) OR (Diphenyl Ethers, Halogenated) OR (Ethers, Halogenated Diphenyl) OR (Chlorinated Diphenyl Ethers) OR (Diphenyl Ethers, Chlorinated) OR (Ethers, Chlorinated Diphenyl) OR (PCDEs) OR (PCDE Compounds) OR (Iodinated Diphenyl Ethers) OR (Diphenyl Ethers, Iodinated) OR (Ethers, Iodinated Diphenyl) OR (Brominated Diphenyl Ethers) OR (Diphenyl Ethers, Brominated) OR (Ethers, Brominated Diphenyl) OR (PBDEs) OR (Polybrominated Diphenyl Ethers) OR (Diphenyl Ethers, Polybrominated) OR (Ethers, Polybrominated Diphenyl) OR (PBDE Compounds) OR (Fluorinated Diphenyl Ethers) OR (Diphenyl Ethers, Fluorinated) OR (Ethers, Fluorinated Diphenyl)

#4 perfluoroalkyl OR polyfluoroalkyl OR perfluorinated OR perfluorooctanoic OR perfluorooctane OR perfluorohexane OR PFOS OR PFOA OR PFHxS OR PFAS*

#5 phthalate OR (mono-n-butyl phthalate) OR (Mono-benzyl phthalate) OR (mono-isobutyl phthalate) OR ( mono-ethyl phthalate) OR (mono-(2-ethyl-5-carboxypentyl) phthalate) OR MBP OR MEP OR DEHP OR MIBP OR MCPP OR MBzP OR PAEs*

#6 #2 OR #3 OR #4 OR #5

#7 #1 AND #6

The last search was run on November 2, 2021.

**2.Search strategy for Embase (Ovid) 105**

#1 'pregnancy diabetes mellitus'/exp OR 'diabetes mellitus gravidarum' OR 'diabetes, gestational' OR 'diabetes, pregnancy' OR 'gestational diabetes' OR 'gestational diabetes mellitus' OR 'pregnancy diabetes' OR 'pregnancy diabetes mellitus' OR 'pregnancy in diabetics'

#2 'polychlorinated biphenyl'/exp OR 'pcb' OR 'phenoclor' OR 'polychlorinated biphenyl' OR 'polychlorinated biphenyl mixture' OR 'polychlorinated biphenyls' OR 'polychlorinated diphenyl' OR 'polychlorobiphenyl' OR 'polychlorodiphenyl'

#3 'poly-brominated diphenyl ethers'/exp OR 'diphenyl ethers, halogenated' OR 'ethers, halogenated diphenyl' OR 'chlorinated diphenyl ethers' OR 'diphenyl ethers, chlorinated' OR 'ethers, chlorinated diphenyl' OR 'pcdes' OR 'pcde compounds' OR 'iodinated diphenyl ethers' OR 'diphenyl ethers, iodinated' OR 'ethers, iodinated diphenyl' OR 'brominated diphenyl ethers' OR 'diphenyl ethers, brominated' OR 'ethers, brominated diphenyl' OR 'pbdes' OR 'polybrominated diphenyl ethers' OR 'diphenyl ethers, polybrominated' OR 'ethers, polybrominated diphenyl' OR 'pbde compounds' OR 'fluorinated diphenyl ethers' OR 'diphenyl ethers, fluorinated' OR 'ethers, fluorinated diphenyl'

#4 'phthalic acid'/exp OR 'mono n butyl phthalate'/exp OR 'mono benzyl phthalate'/exp OR 'mono isobutyl phthalate'/exp OR 'mono ethyl phthalate'/exp OR 'mono 2 ethyl 5 carboxypentyl phthalate'/exp

#5 'perfluoroalkyl substance' OR 'polyfluoroalkyl substance' OR 'perfluoro compound' OR 'perfluorooctanoic acid' OR 'perfluorooctanesulfonic acid' OR 'perfluorohexanesulfonic acid' OR pfos OR pfoa OR pfhxs OR pfas*

#6 #2 OR #3 OR #4 OR #5

#7 #1 AND #6

**3.Search strategy for Web of Science 144**

#1 TOPIC: (Diabetes, Pregnancy-Induced) OR (Diabetes, Pregnancy Induced) OR (Pregnancy-Induced Diabetes) OR (Gestational Diabetes*) OR (Diabetes Mellitus, Gestational) OR (Gestational Diabetes Mellitus) OR GDM

#2 TOPIC: (polychlorinated biphenyls) OR (Biphenyls, Polychlorinated) OR (Polychlorobiphenyl Compounds) OR (Compounds, Polychlorobiphenyl) OR (Polychlorinated Biphenyl) OR (Biphenyl, Polychlorinated) OR PCBs

#3 TOPIC: (poly-brominated diphenyl ethers [MeSH]) OR (Diphenyl Ethers, Halogenated) OR (Ethers, Halogenated Diphenyl) OR (Chlorinated Diphenyl Ethers) OR (Diphenyl Ethers, Chlorinated) OR (Ethers, Chlorinated Diphenyl) OR (PCDEs) OR (PCDE Compounds) OR (Iodinated Diphenyl Ethers) OR (Diphenyl Ethers, Iodinated) OR (Ethers, Iodinated Diphenyl) OR (Brominated Diphenyl Ethers) OR (Diphenyl Ethers, Brominated) OR (Ethers, Brominated Diphenyl) OR (PBDEs) OR (Polybrominated Diphenyl Ethers) OR (Diphenyl Ethers, Polybrominated) OR (Ethers, Polybrominated Diphenyl) OR (PBDE Compounds) OR (Fluorinated Diphenyl Ethers) OR (Diphenyl Ethers, Fluorinated) OR (Ethers, Fluorinated Diphenyl)

#4 TOPIC: perfluoroalkyl OR polyfluoroalkyl OR perfluorinated OR perfluorooctanoic OR perfluorooctane OR perfluorohexane OR PFOS OR PFOA OR PFHxS OR PFAS*

#5 TOPIC: phthalate OR (mono-n-butyl phthalate) OR (Mono-benzyl phthalate) OR (mono-isobutyl phthalate) OR ( mono-ethyl phthalate) OR (mono-(2-ethyl-5-carboxypentyl) phthalate) OR MBP OR MEP OR DEHP OR MIBP OR MCPP OR MBzP OR PAEs*

#6 #2 OR #3 OR #4 OR #5

#7 #1 AND #6
