# Supplementary material for: Endocrine-disrupting chemicals and the risk of gestational diabetes mellitus: a systematic review and meta-analysis
Source: Environ Health. 2022 May 16;21:53. doi: 10.1186/s12940-022-00858-8 (PMC9109392; doi:10.1186/s12940-022-00858-8)
Supplement: Supplementary file 3 — Additional file 3: Table 2. Quality assessment of the included studies. [file 12940_2022_858_MOESM3_ESM.doc]

Endocrine-disrupting chemicals and the risk of gestational diabetes mellitus: A systematic review and meta-analysis

Dandan Yan^1^, Yang Jiao^2^, Honglin Yan^1^, Tian Liu^1^, Hong Yan^2^, Jingping Yuan^1^

^1^Department of Pathology, Renmin Hospital of Wuhan University, 238 Jiefang-Road, Wuchang District, Wuhan, 430060, P.R. China. ^2^Department of Health Toxicology, MOE Key Lab of Environment and Health, School of Public Health, Tongji Medical College, Huazhong University of Science and Technology, 13 Hangkong-Road, Wuhan, 430030, P.R. China.

***Corresponding authors**: Jingping Yuan, PhD

Department of Pathology, Renmin Hospital of Wuhan University, 238 Jiefang-Road, Wuchang District, Wuhan, 430060, PR China.

Email: yuanjingping@whu.edu.cn (JP. Yuan) Tel: +86-027-88041911-85523.

Table 2 Quality assessment of the included studies

| **Study design** | **Study** | **Selection** | | | | **Comparability** | | **Outcome** | | | **Quality score** |
| --- | --- | --- | --- | --- | --- | --- | --- | --- | --- | --- | --- |
|  |  | **Item 1** | **Item 2** | **Item 3** | **Item4** | **Item 5a** | **Item 5b** | **Item 6** | **Item7** | **Item 8** |  |
| Cohort study | Zhang et al. 2015 | 1 | 1 | 1 | 1 | 1 | 0 | 0 | 1 | 1 | 7 |
|  | Vafeidi et al.2016 | 1 | 1 | 1 | 1 | 1 | 1 | 1 | 1 | 1 | 9 |
|  | Shapiro et al. 2015 | 1 | 1 | 1 | 1 | 1 | 0 | 1 | 1 | 1 | 8 |
|  | Shapiro et al. 2016 | 1 | 1 | 1 | 1 | 1 | 1 | 1 | 1 | 1 | 9 |
|  | Jaacks et al. 2016 | 1 | 1 | 1 | 1 | 1 | 1 | 1 | 1 | 1 | 9 |
|  | Rahman et al.2019 | 1 | 1 | 1 | 1 | 1 | 1 | 1 | 1 | 1 | 9 |
|  | M-Santander et al. 2017 | 1 | 1 | 1 | 1 | 1 | 0 | 1 | 1 | 1 | 8 |
|  | Valvi et al. 2017 | 1 | 1 | 1 | 1 | 1 | 0 | 0 | 1 | 1 | 7 |
|  | Shaffer et al.2019 | 1 | 1 | 1 | 1 | 0 | 0 | 1 | 1 | 1 | 7 |
|  | Smarr et al. 2016 | 1 | 1 | 1 | 1 | 1 | 0 | 0 | 1 | 1 | 7 |
|  | Zhang et al. 2017 | 1 | 1 | 1 | 1 | 1 | 0 | 1 | 1 | 1 | 8 |
|  | Wang et al.2018b | 1 | 1 | 1 | 1 | 1 | 0 | 1 | 1 | 1 | 8 |
|  | Gao et al. 2021 | 1 | 1 | 1 | 1 | 1 | 1 | 1 | 1 | 1 | 9 |
|  | Zukin et al. 2021 | 1 | 1 | 1 | 1 | 1 | 0 | 1 | 1 | 1 | 8 |
|  | Yu et al.2021 | 1 | 1 | 1 | 1 | 1 | 0 | 1 | 1 | 1 | 8 |
|  | Preston et al.2020 | 1 | 1 | 1 | 1 | 1 | 0 | 1 | 1 | 1 | 8 |
| Case-control study | Eslami et al.2016 | 1 | 1 | 1 | 1 | 1 | 1 | 1 | 1 | 1 | 9 |
|  | Wang et al.2018a | 1 | 1 | 1 | 1 | 1 | 0 | 1 | 1 | 1 | 8 |
|  | Zhang et al.2018 | 1 | 1 | 1 | 1 | 0 | 0 | 1 | 1 | 1 | 7 |
|  | Liu et al.2018 | 1 | 1 | 1 | 1 | 1 | 0 | 1 | 1 | 1 | 8 |
|  | Fisher et al.2018 | 1 | 1 | 0 | 1 | 1 | 0 | 1 | 1 | 1 | 7 |
|  | Liu et al. 2019 | 1 | 1 | 1 | 1 | 1 | 1 | 1 | 1 | 1 | 9 |
|  | Xu et al. 2020 | 1 | 1 | 1 | 1 | 1 | 0 | 1 | 1 | 1 | 8 |
| Cross-sectional study | Guo et al. 2020 | 1 | 1 | 1 | 1 | 1 | 0 | 0 | 1 | 1 | 7 |
|  | Neblett et al. 2020 | 0 | 1 | 1 | 1 | 1 | 0 | 1 | 1 | 1 | 7 |

The study quality was assessed according to the Newcastle Ottawa Quality assessment scale. This scale awards a maximum of 9 points to each study, 1 = “Yes”, 0 = “No”, “Unable to determine” or “Not available”. For cohort study, Item 1: Representativeness of the exposed cohort; Item 2: Selection of the non-exposed cohort; Item 3: Ascertainment of exposure; Item 4: Demonstration that outcome of interest was not present at start of study; Item 5a, Comparability of cohorts on the basis of the design or analysis; Item 5b, cohorts comparable on other factor(s); Item 6, Assessment of outcome; Item 7: Was follow-up long enough for outcomes to occur; Item 8, Adequacy of follow up of cohorts. For case-control and cross-sectional studies: Item 1: Is the case definition adequate? Item 2: Representativeness of the cases; Item 3: Selection of Controls; Item 4: Definition of Controls; Item 5a: Comparability of cases and controls on the basis of the design or analysis; Item 5b, comparable on other factor(s); Item 6: Ascertainment of exposure; Item 7: Same method of ascertainment for cases and controls; Item 8: Non-Response rate.
