# Supplementary material for: Endocrine-disrupting chemicals and the risk of gestational diabetes mellitus: a systematic review and meta-analysis
Source: Environ Health. 2022 May 16;21:53. doi: 10.1186/s12940-022-00858-8 (PMC9109392; doi:10.1186/s12940-022-00858-8)
Supplement: Supplementary file 5 — Additional file 5: Figure 1. funnel plot of PCBs (A), PBDEs(B), PAEs(C), and PFAS (D) exposure and risk of GDM. [file 12940_2022_858_MOESM5_ESM.docx]

Responses to reviewers

Dear Editors and Reviewers,

Thank you for your letter and the reviewers’ comments regarding our manuscript entitled “Endocrine-disrupting chemicals and the risk of gestational diabetes mellitus: A systematic review and meta-analysis”, which was submitted to *Environmental Health* for publication. These comments are all valuable and helpful for revising and improving our paper. We are very pleased to revise the manuscript and answer the reviewers’ questions to make the paper more acceptable. Revised portions are marked in red in this revised manuscript.

The responses to these comments are as following:

Reviewer #1:

This systematic review and meta-analysis by Yan et al shows that EDCs exposure may be a risk factor for gestational diabetes mellitus. The subject is interesting and timely and the analysis seems to be well done. The paper needs to be edited by an English-speaking person.

Specific points

Line 67, reference 10 is specific of gestational diabetes, a more general article would fit better here, i.e. Gore et al, Endo Rev 2015 or Heindel et al, Rep Toxicol 2017.

***Author reply***

Thanks for reviewer’s opinions and suggestions.

Your suggestion is greatly appreciated. After consulting these two articles [1, 2], we think your recommendation is more appropriate. The reference 10 has been changed to Gore et al, Endo Rev 2015 and Heindel et al, Rep Toxicol 2017.

Line 70, specify "various classes", here are phthalates, bisphenol-a and metals.

***Author reply***

Thanks so much for your careful review on our manuscript.

We have changed the sentence “Previous studies have indicated that exposure to EDCs of various classes may be closely associated with the occurrence and development of GDM” to “Previous studies have indicated that exposure to EDCs including phthalates, bisphenol-A and metals may be closely associated with the occurrence and development of GDM”. The revised portions are marked in red in this revised manuscript.

Line 99, please add language restrictions if applicable.

***Author reply***

Thanks for reviewer’s opinions and suggestions. We have added language restrictions in the revised manuscript.

We’ve recognized that some of the descriptions in the previous copy were really not so accurate and a little bit arbitrary due to our poor English level. We have handed over our manuscript to an English-speaking person for language editing.

Line 115, include initial of these two researchers. Are they the same as in line 102? The same in line 120.

Include in data extraction and quality assessment how disagreements between the two researchers were solved.

***Author reply***

Thanks for the reviewer’s comments.

In line 115 and line 120, the two investigators are the same as in line 102. The two investigators were DDY and YJ, we have added the two investigators’ name in the revised manuscript.

During data extraction and quality assessment, any discrepancies were resolved by a joint reevaluation (JPY and HY) of the study. We have added these details in the revised manuscript.

Line 142, studies or articles instead of researches?

***Author reply***

I am sorry to say it’s really a spelling mistake, we have changed the word “researches” to “articles” in the revised manuscript. Thank you for your carefulness and reminding us of the improper report.

Figure 1, study selection. Records screened should be 152 from which 45 were excluded and then, 107 were assessed for eligibility. Please, briefly explain in the text why these 45 articles were not pertinent.

***Author reply***

Thanks for the reviewer’s comments.

After screening the titles and abstracts, 45 articles were excluded for the following reasons: did not meet the selection criteria, irrelevant exposure or outcome, or studies not related to systematic review. We have added these reasons in the revised manuscript accordingly.

Line 171 delete, EDCs of

In most cases the number of eligible articles were lower than 10, sometimes only 4 (PBDEs and risk of GDM). Please comment whether this could generate a potential publication bias.

Line 299 "…PAE-induced estrogen…" This needs to be re-written

***A******uthor reply***

Thanks for reviewer’s opinions and suggestions.

We deeply apologize for our careless in writing the paper.

In line 171, the “EDCs of” has been deleted. In line 299, the sentence “…PAE-induced estrogen…” has been changed to “the prolonged activation of estrogen receptor ERα by the environmental estrogen could lead to excess insulin release”.

In addition to Egger’s test[3] and Begg’s test[4], the funnel plot is a simple and intuitive method to judge whether publication bias exists or not. In our present study, the the funnel plots of PCBs, PBDEs, PAEs, and PFAS are shown as following (figure 1). Visual inspection of the funnel plots did not show remarkable asymmetry in all of our results. For example, for PBDEs, it should be noted that, although there were only 4 original studies, 4 articles with 22 reports were included according to PBDE congeners.

Your suggestions greatly inspired us and are greatly appreciated. Due to the limited number of included studies, we were unable to perform a more comprehensive analysis. In our future study, we will continue to update this study and search for more original articles for meta-analysis. We deeply appreciate your reconsideration of our revised manuscript.

Figure 1. funnel plot of PCBs (A), PBDEs(B), PAEs(C), and PFAS (D) exposure and risk of GDM.

Reviewer 2

EDC and GDM review -- The potential impact of exposure to EDC in pregnancy is an important topic. The current review focuses on a subset of EDCs (PBDE, PAE, PFAS) in relation to a single pregnancy outcome, namely GDM. Despite the potential public health impacts, compelling evidence of an association remains limited

-	I am not qualified to assess statistical aspects of the analysis so will leave this to others

-	Generally speaking the study is sound, though the background is missing much of the evidence in the field, summarised nicely in several recent articles, including Lancet Diabetes Endocrinol. 2020 Aug; 8(8): 703-718. The overall effect sizes reported are small, particularly for cohort studies. Given the confidence intervals, the strength of overall evidence is weak and this should be discussed. Figure 4 in particular does not show a strong relationship.

***Author reply***

Thank the reviewer for the comments and suggestions.

Your suggestion is greatly appreciated. Your suggestion is greatly appreciated. After consulting more references[5, 6] and combining the existing results of our present study, we have recapitulates the evidence already linking EDC to increased GDM risk in the Discussion part, the revised portions are marked in red in this revised manuscript. We hope the revised paper to be more reasonable and convincing. We deeply appreciate your reconsideration of our revised manuscript.

Strengths

-	Very important area of research with potentially profound public health implications

-	Large final sample size of nearly 24,000 participants

-	Overall, the manuscript is well structured and an important addition to the literature

-	Inclusion of studies with direct measures of EDC is an important criteria that increases confidence that exposure measures are reasonably accurate

Issues for consideration

-	Overall, the manuscript would benefit from the expertise of a professional editor to correct the numerous minor grammatical errors

***Author reply***

Thank the reviewer for the comments.

We’ve recognized that some of the descriptions in the previous copy were really not so accurate and a little bit arbitrary due to our poor English level. We have handed over our manuscript to an English-speaking person for language editing. The revised portions are marked in red in this revised manuscript. We deeply appreciate your reconsideration of our revised manuscript.

-	The authors state that the incidence of GDM is increasing markedly, but this is only in certain countries and the relative incidence is highly variable. This should be included in the introduction

***Author reply***

Thank you for reminding us the improper description on the study.

We’ve recognized that some of the descriptions in the previous copy were really not so accurate. Your suggestions greatly inspired us and are greatly appreciated. In the section of “introduction”, we have supplemented more information about the incidence of GDM.

The sentence “The incidence of GDM widely varies depending on the diagnostic criteria used and population characteristics[7, 8]. The prevalence of GDM is increasing, which is linked to an increase in maternal obesity in recent decades, and it affects 6% – 25% of pregnant women (depending on the diagnostic criteria)[7]”. We hope that this revised manuscript will be convincing.

-	The adverse outcomes of GDM include macrosomia and altered metabolic status of progeny and this is potentially associated with early onset T2D in offspring. Please add a little more to the intro

***Author reply***

Thanks so much for your careful review on our manuscript.

Your suggestion is greatly appreciated. We have added more information about the adverse outcomes for the mother and fetus in the introduction. Details are as follows “Evidences indicated that GDM is associated with dramatic adverse health effects for the mother and their offspring. Fetuses born to mothers with GDM are at an increased risk of multiple complications, including macrosomia, birth injury, altered metabolic status, neonatal hypoglycemia, respiratory distress, and type 2 diabetes mellitus (T2DM), later in life[9-14]. Meanwhile women with GDM are more likely to develop gestational hypertension, preeclampsia, caesarean section, and shoulder dystocia, among other serious complications[9, 10, 13, 14].”

The revised portions are marked in red in this revised manuscript. We deeply appreciate your reconsideration of our revised manuscript.

-	Although the authors have done their best to factor in study heterogeneity and publication bias, these remain problematic in terms of generating compelling findings

***Author reply***

Thanks for reviewer’s opinions and suggestions.

Your suggestions greatly inspired us and are greatly appreciated. For publication bias, in addition to Egger [3] and Begg tests[4], the funnel plot is a simple and intuitive method to judge whether publication bias exists or not. In our present study, the the funnel plots of PCBs, PBDEs, PAEs, and PFAS are shown in figure 1 (showing above). Visual inspection of the funnel plots did not show remarkable asymmetry in all of our results.

For study heterogeneity, due to the limited number of included studies, we are unable to perform additional subgroup analyses to explore the sources of heterogeneity. Your comment is very helpful and instructive for us. We have recognized and discussed this key limitations of our present study in the revised manuscript. We hope the revised paper to be more reasonable and convincing. In our future study, we will continue to update this study and search for more original articles for meta-analysis. We deeply appreciate your reconsideration of our revised manuscript.

-	There is insufficient summary of the evidence already linking EDC to increased GDM risk.

***Author reply***

Thank you for reminding us the insufficient description on the study.

Your suggestion is greatly appreciated. After consulting more references (Linda et al. 2020; Lind PM et al. 2018; Gingrich J et al.2020)[5, 6, 15] and combining the existing results of our present study, we have recapitulates the evidence already linking EDC to increased GDM risk in the Discussion part, the revised portions are marked in red in this revised manuscript. We hope the revised paper to be more reasonable and convincing.

-	L241 - The statement 'the potential link between EDCS and GDM risk remains obscure' does not adequately take into account the existing evidence

***Author reply***

Thanks so much for your careful review on our manuscript.

We’ve recognized that some of the descriptions in the previous copy were really not so accurate and a little bit arbitrary. Your suggestions greatly inspired us and are greatly appreciated. After consulting more relevent references, we have recapitulates the evidence already linking EDC to increased GDM risk in the Discussion part, the revised portions are marked in red in this revised manuscript. We hope the revised paper to be more reasonable and convincing.

Minor points

-	L69 - more detail on 'postnatal pathologies'70

-	L70 - more detail on which specific EDC implicated in GDM

***Author reply***

Thanks for reviewer’s opinions and suggestions.

In line 70, we have added the detail on “postnatal pathologies” in the revised manuscript.

In line 70, we have replaced the “various classes” to “phthalates, bisphenol-A and metals” in the revised manuscript.

We have added more detail on “postnatal pathologies” and “ which specific EDC implicated in GDM” in line 69 and 70,

specify "various classes", here are phthalates, bisphenol-a and metals.

***Author reply***

Thanks so much for your careful review on our manuscript.

We have changed the sentence “Previous studies have indicated that exposure to EDCs of various classes may be closely associated with the occurrence and development of GDM” to “Previous studies have indicated that exposure to EDCs including phthalates, bisphenol-A and metals may be closely associated with the occurrence and development of GDM”. The revised portions are marked in red in this revised manuscript.

-	L80 - this is not a comprehensive review of all EDC. Only a subset of EDC and only 1 outcome is considered.

***Author reply***

Thank you for your carefulness and reminding us of the improper report.

Your comment is very helpful and instructive for us. Due to the insufficient number of relevant studies, the types of EDCs and complications of pregnancy enrolled in this study are limited.

We have recognized and discussed this key limitations of our present study in the revised manuscript. We hope the revised paper to be more reasonable and convincing. In our future study, we will continue to update this study and search for more original articles for meta-analysis. We deeply appreciate your reconsideration of our revised manuscript.

**References:**

1.	Heindel JJ, Blumberg B, Cave M, Machtinger R, Mantovani A, Mendez MA, Nadal A, Palanza P, Panzica G, Sargis R *et al*: **Metabolism disrupting chemicals and metabolic disorders**. *REPROD TOXICOL* 2017, **68**:3-33.

2.	Gore AC, Chappell VA, Fenton SE, Flaws JA, Nadal A, Prins GS, Toppari J, Zoeller RT: **Executive Summary to EDC-2: The Endocrine Society's Second Scientific Statement on Endocrine-Disrupting Chemicals**. *ENDOCR REV* 2015, **36**(6):593-602.

3.	Egger M, Davey SG, Schneider M, Minder C: **Bias in meta-analysis detected by a simple, graphical test**. *BMJ* 1997, **315**(7109):629-634.

4.	Begg CB, Mazumdar M: **Operating characteristics of a rank correlation test for publication bias**. *BIOMETRICS* 1994, **50**(4):1088-1101.

5.	Kahn LG, Philippat C, Nakayama SF, Slama R, Trasande L: **Endocrine-disrupting chemicals: implications for human health**. *Lancet Diabetes Endocrinol* 2020, **8**(8):703-718.

6.	Gingrich J, Ticiani E, Veiga-Lopez A: **Placenta Disrupted: Endocrine Disrupting Chemicals and Pregnancy**. *Trends Endocrinol Metab* 2020, **31**(7):508-524.

7.	Sacks DA, Hadden DR, Maresh M, Deerochanawong C, Dyer AR, Metzger BE, Lowe LP, Coustan DR, Hod M, Oats JJ *et al*: **Frequency of gestational diabetes mellitus at collaborating centers based on IADPSG consensus panel-recommended criteria: the Hyperglycemia and Adverse Pregnancy Outcome (HAPO) Study**. *DIABETES CARE* 2012, **35**(3):526-528.

8.	Engelgau MM, Herman WH, Smith PJ, German RR, Aubert RE: **The epidemiology of diabetes and pregnancy in the U.S., 1988**. *DIABETES CARE* 1995, **18**(7):1029-1033.

9.	Johns EC, Denison FC, Norman JE, Reynolds RM: **Gestational Diabetes Mellitus: Mechanisms, Treatment, and Complications**. *Trends Endocrinol Metab* 2018, **29**(11):743-754.

10.	Metzger BE, Lowe LP, Dyer AR, Trimble ER, Chaovarindr U, Coustan DR, Hadden DR, McCance DR, Hod M, McIntyre HD *et al*: **Hyperglycemia and adverse pregnancy outcomes**. *N Engl J Med* 2008, **358**(19):1991-2002.

11.	Burlina S, Dalfra MG, Lapolla A: **Short- and long-term consequences for offspring exposed to maternal diabetes: a review**. *J Matern Fetal Neonatal Med* 2019, **32**(4):687-694.

12.	Damm P, Houshmand-Oeregaard A, Kelstrup L, Lauenborg J, Mathiesen ER, Clausen TD: **Gestational diabetes mellitus and long-term consequences for mother and offspring: a view from Denmark**. *DIABETOLOGIA* 2016, **59**(7):1396-1399.

13.	Farahvar S, Walfisch A, Sheiner E: **Gestational diabetes risk factors and long-term consequences for both mother and offspring: a literature review**. *Expert Rev Endocrinol Metab* 2019, **14**(1):63-74.

14.	Billionnet C, Mitanchez D, Weill A, Nizard J, Alla F, Hartemann A, Jacqueminet S: **Gestational diabetes and adverse perinatal outcomes from 716,152 births in France in 2012**. *DIABETOLOGIA* 2017, **60**(4):636-644.

15.	Lind PM, Lind L: **Endocrine-disrupting chemicals and risk of diabetes: an evidence-based review**. *DIABETOLOGIA* 2018, **61**(7):1495-1502.
